# A long‐term study of size variation in Northern Goshawk *Accipiter gentilis* across Scandinavia, with a focus on Norway

**DOI:** 10.1002/ece3.10789

**Published:** 2023-12-07

**Authors:** Samuel J. Walker, Terje Lislevand, Hanneke J. M. Meijer

**Affiliations:** ^1^ Department of Natural History, University Museum of Bergen University of Bergen Bergen Norway; ^2^ Department of Biosciences, Centre for Ecological and Evolutionary Synthesis (CEES) University of Oslo Oslo Norway; ^3^ Human Origins Program, Department of Anthropology National Museum of Natural History, Smithsonian Institution Washington District of Columbia USA

**Keywords:** climate change, hawks, medieval, metrics, Scandinavia

## Abstract

Changing climate and growing human impacts are resulting in globally rising temperatures and the widespread loss of habitats. How species will adapt to these changes is not well understood. The Northern Goshawk (*Accipiter gentilis*) can be found across the Holarctic but is coming under more intense pressure in many places. Studies of recent populations in Finland and Denmark have shown a marked decline in body size of Northern Goshawks over the past century. Here we investigate long‐term changes to Norwegian populations of Northern Goshawk by including material from the Middle Ages. We measured 240 skeletons of modern Northern Goshawks from Norway, Sweden, Denmark and Finland, and 89 Medieval Goshawk bones. Our results show that Norwegian and Swedish female Goshawks have decreased in size over the past century, whilst males showed little decline. Medieval female Goshawks were larger than contemporary females. A decline in forest habitats and a concomitant shift towards smaller prey likely drove a shift to smaller body size in Northern Goshawks. Our study shows that significant body size changes in birds can occur over relatively short time spans in response to environmental factors, and that these effects can sometimes differ between sexes.

## Graphical abstract

Here we investigate long‐term changes to Scandinavian populations of Northern Goshawk (*Accipiter gentilis*). Our results show that Norwegian and Swedish female Goshawks have decreased in size over the past century, whilst males showed little decline. Furthermore, Norwegian Medieval female Goshawks were larger than contemporary females. Our study shows that significant body size changes in birds can occur over relatively short time spans in response to environmental factors, and that these effects can sometimes differ between sexes.

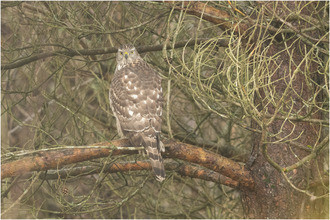

## 1 INTRODUCTION

An organism's body size is considered a key trait linked to other life‐history traits, dispersal ability, population dynamics and ecosystem function (Peters & Wassenberg, 1983). A number of bird species have undergone body size changes during the 20th century. These changes are varied with some species increasing (Kaňuščák et al., 2004; Moreno‐Rueda & Rivas, 2007) but the majority showing a decrease in overall body size (Jakober & Stauber, 2000; Riddell et al., 2019; Weeks et al., 2020, 2022; Yom‐Tov & Yom‐Tov, 2006). This overall decrease, seen not only in birds, has led to some suggesting it is a possible global response to climate change (Daufresne et al., 2009; Gardner et al., 2011; Ryding et al., 2021; Sheridan & Bickford, 2011). However, others have suggested there is not enough of a uniform decline in size to back up these claims (Riemer et al., 2018; Siepielski et al., 2019; Teplitsky & Millien, 2014). The reasons behind body size changes are often varied and complex; changes in food availability and diet, competition for resources, response to temperature and shifts in distribution have all been argued to play a role. All the studies above agree that more deep‐time evidence is needed to better understand future changes in species and the reasons for changes during the 20th century (Millien et al., 2006). Nevertheless, there are few deep‐time studies of body size in birds, especially within Fennoscandia (i.e. Ericson, 1989; Stewart, 2007; Walker & Meijer, 2021).

**FIGURE 1:**
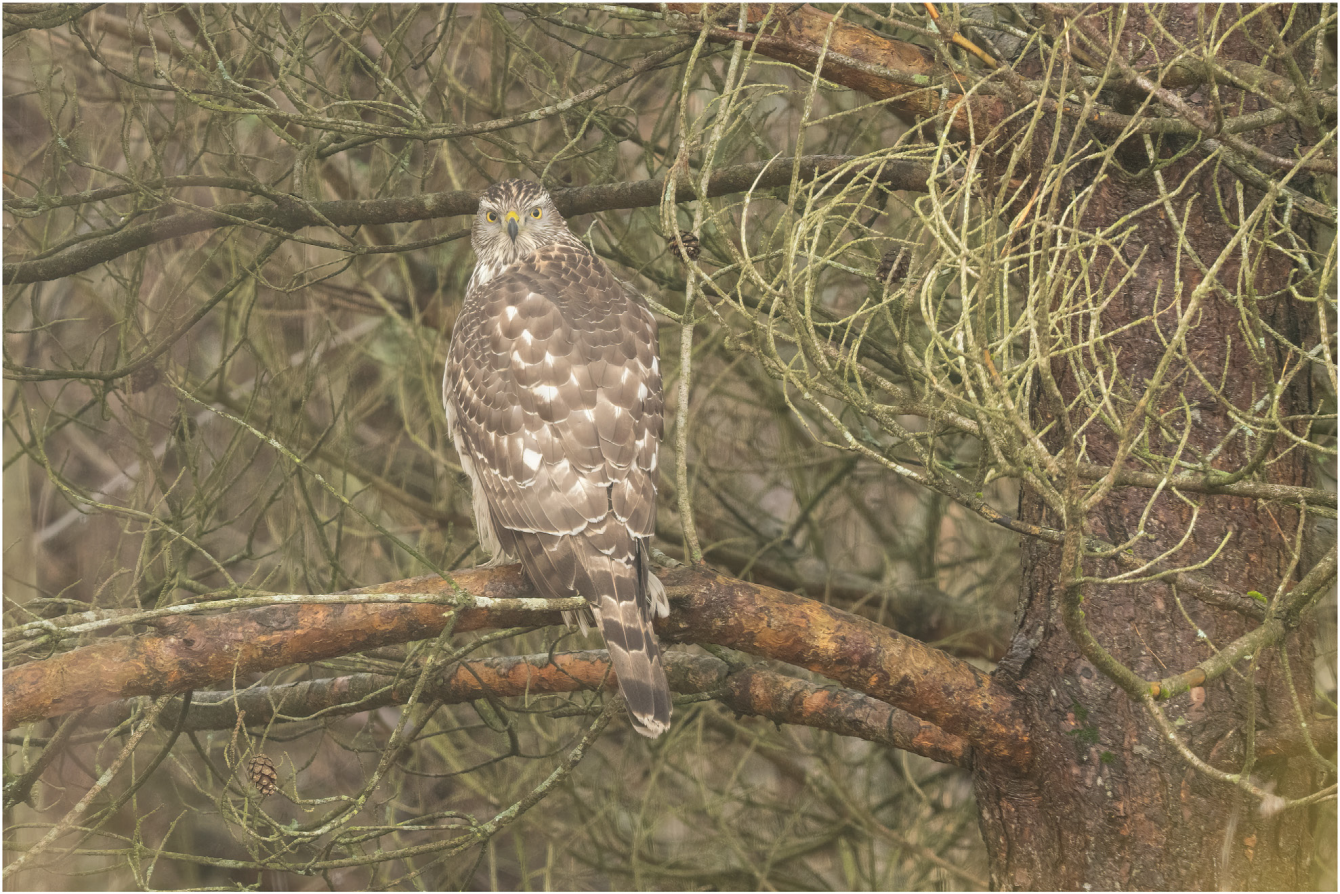
Image of a juvenile Northern Goshawk (*Accipiter gentilis*) taken on the 15th of January 2023, Klokkarvik, Sotra, Norway. Photograph taken by Bjørn Reidar Olsson, University Museum of Bergen.

Here we provide long‐term body size data on the Northern Goshawk (*Accipiter gentilis*; Figure 1), a species widely distributed across the Palearctic and Nearctic regions. Although it is not currently regarded as a globally threatened species (IUCN, 2021), many national populations are declining, for example in Fennoscandia (Artsdatabanken, 2021; Den Danske Rødliste, 2023; Red List of Finnish Species, 2019; The Swedish Taxonomy Initiative, 2023). The species inhabits mature woodland (Squires et al., 2020), a habitat which is under threat and decreasing across the globe (BirdLife International, 2022). The overall population trend of *A. gentilis* is poorly understood. In Europe it is considered that numbers are decreasing at <25% over 21 years (3 generations; BirdLife International, 2015). The main threats to Northern Goshawks are deforestation, persecution, nest robbing for falconry, pesticides (Squires et al., 2020) and the development of wind farms (STRIX, 2012). Forested areas are widespread in the Nordic countries (especially Finland and Sweden); however, these habitats are at risk as deforestation remains an issue. The success of Northern Goshawk populations is heavily reliant upon suitable nesting‐sites and prey availability (Rutz et al., 2006). This is the case for Norwegian Northern Goshawk populations, which are dependent upon Grouse species, such as Western Capercaillie (*Tetrao urogallus*), Black Grouse (*Tetrao tetrix*), Hazel Grouse (*Bonasa bonasia*) and Willow Grouse (*Lagopus lagopus*; Höglund, 1964; Huhtala, 1976; Lindén & Wikman, 1983; Selås, 1989; Tornberg, 1997; Widén, 1987).

As in other raptors (Székely et al., 2007) Northern Goshawks show reversed sexual dimorphism where females are larger than males. Several studies have highlighted recent changes in *A. gentilis* body size. A study in Denmark identified a decrease in body size of Danish *A. gentilis* during the 20th century, likely related to diet and a shift in food availability (Yom‐Tov & Yom‐Tov, 2006). This size decrease was more evident in females and juveniles than in males. In Finland, research on modern populations dating between 1962 and 2008 showed a decrease in body size in both sexes. This was interpreted to reflect a change in prey species (Tornberg et al., 2014), but the authors also note that climate change may be a contributing factor. Interestingly, Tornberg et al. (2014) also identified morphological changes to the sternum, whereby Goshawks were displaying a more *Buteo*‐type sternum, characterised by a relatively short keel, allowing for a lighter wing loading. This morphology is typical in raptors with a broader diet (Jaksić & Carothers, 1985; Jenkins, 1995). In the case of the Finnish Goshawks, this reflects a shift from fast moving prey such as grouse, to more slow‐moving corvids, gulls and larger mammalian prey such as hares (Tornberg et al., 2014). This suggests that Goshawks are shifting from an ambush raptor (like *Falco* and *Accipiter* spp.) to more of a searching raptor (such as *Buteo* and *Aquila* spp.). The work by Tornberg et al. (2014) and Yom‐Tov and Yom‐Tov (2006) has highlighted the ability of recent populations of *A. gentilis* to adapt to environmental change. However, to the best of our knowledge, there is currently no deep‐time studies of size change in Northern Goshawks. Within our paper, we identify skeletal changes in modern and archaeological (Medieval) populations of *A. g. gentilis*, including Swedish and Norwegian data alongside new data for Finland and Denmark.

## 2 METHODS

### 2.1 Modern comparative material

To analyse changes in both modern and past populations of *A. gentilis*, metric data of the nominate *Accipiter gentilis gentilis* were collected from across the Nordic countries (Norway, Sweden, Denmark and Finland). The Northern Goshawk is a sedentary species, generally choosing to breed and winter in the same area. There are some exceptions to this in North America, Fennoscandia and Russia (Squires et al., 2020). However, individuals from Fennoscandia rarely migrate further than 300 km (Squires et al., 2020). The nominate *A. g. gentilis* is distributed across Europe and east to the Urals, Caucasus and Asia Minor, and southwards to NW Africa (Squires et al., 2020). The slightly larger subspecies *Accipiter gentilis buteoides* breeds in northern Fennoscandia and Siberia, wintering in south and central Eurasia (Ferguson‐Lees & Christie, 2005). Goshawks found in northern Finland and northern Sweden likely represent the subspecies *A. g. buteoides* (Gladkov, 1941; Vaurie, 1965). Therefore, we excluded any material from these areas in our study. In addition, young Northern Goshawk from interior parts of Scandinavia are known to winter along the Norwegian coast in the north (Bakken et al., 2003; Fransson & Pettersson, 2001). To avoid mixing of the larger subspecies *A. g. buteoides* within the modern comparative sample, we decided to use material from the south of Norway, Sweden and Finland.

**FIGURE 2:**
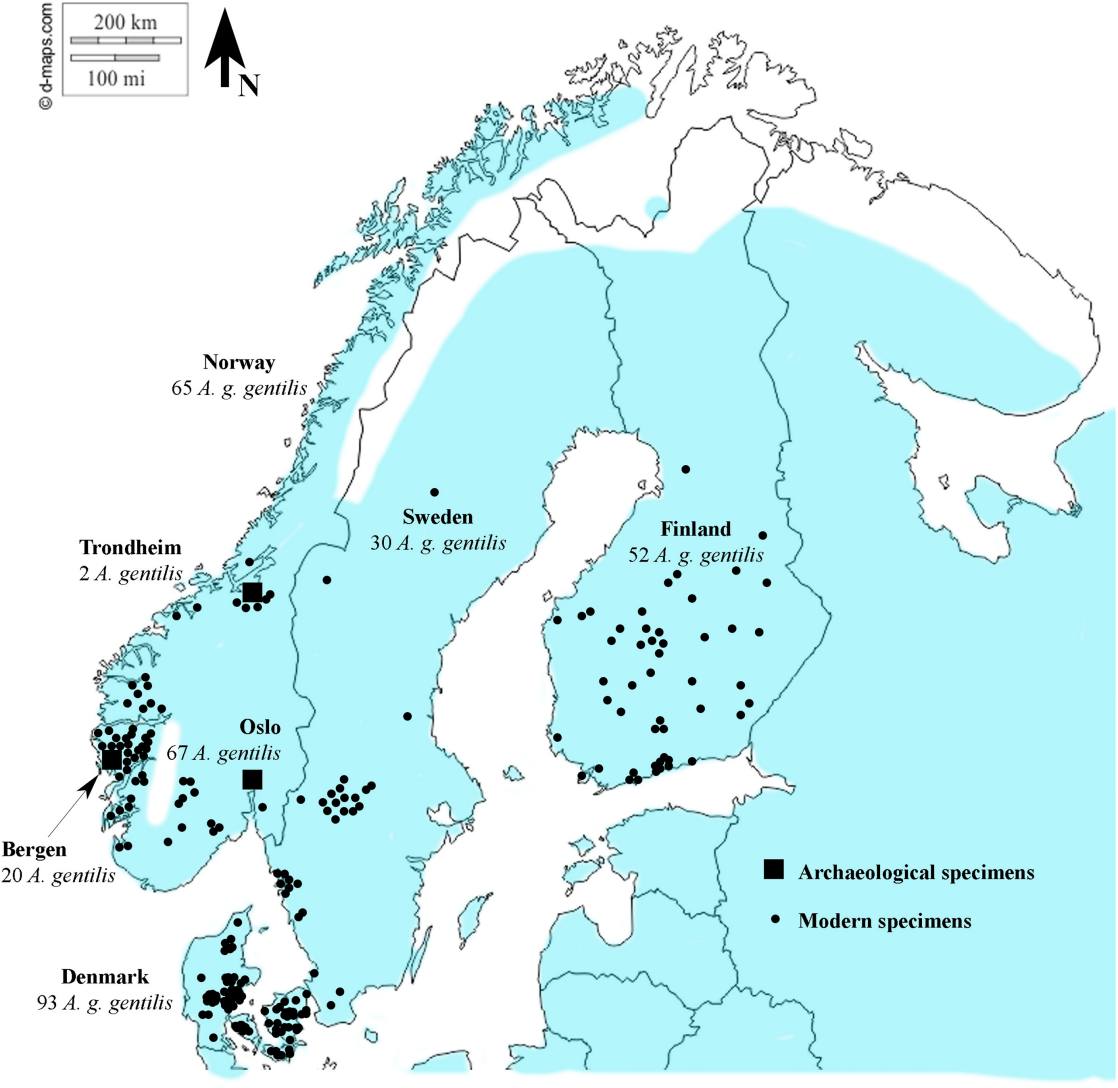
Map showing the general distribution of Northern Goshawk across Scandinavia (blue shaded area), taken from Squires et al. (2020); distribution is not as continuous as suggested here in the mountainous areas (see Gjershaug et al., 1994, for detailed distribution in Norway). The archaeological sites (Oslo, Trondheim and Bergen) and the locations of the comparative *Accipiter gentilis gentilis* specimens are also marked on the map.

Modern comparative skeletons (collected over the past c. 150 years from 1861 to 2015) have been measured from the University Museum of Bergen, the Natural History Museum of Denmark and the Finnish Museum of Natural History (see Appendix 1). The specimens were originally collected from Norway (*n* = 65), Sweden (*n* = 30), Denmark (*n* = 93) and Finland (*n* = 52) (see Figure 2). We measured all skeletal elements, except for the vertebrae and ribs of 240 modern partial and complete *Accipiter gentilis gentilis* skeletons of which 103 were female and 137 were male. It is not clear how sex of the specimens was initially determined; however, we presume that for the majority it was through internal inspection. *Accipiter gentilis* are highly sexually dimorphic, with the female being larger than the male (i.e. reversed sexual size dimorphism). There is very little to no overlap between the sexes of Northern Goshawk (Kenward, 2006). As a result, it was possible to identify 5 modern specimens which were likely to have been mis‐sexed at the time of collection. Wrongly identified sex can be common in *Accipiter* subspecies and especially in *Accipiter gentilis*, as the paired ovaries can sometimes be mistaken for testes (Storer, 1966). The specimens which were wrongly sexed have been reclassified as the correct sex based on our osteological analysis and included within this study. The museum numbers of mis‐sexed specimens are as follows: B 4462 and KL 31303 (originally recorded as males but fall within the female size range and have been reclassified as females), B 9016, NHMD 306590 and NHMD 306670 (originally recorded as females but fall within the male size range and have therefore been reclassified as males). All specimens were measured by SJW using digital callipers. Measurements followed the conventions set out in Von den Driesch (1976). Three additional measurements were recorded: the smallest depth of the distal shaft of the humerus (KB) found in Kraft (1972), depth of the ulna proximal end (Tp) and height of the symphysis of the carpometacarpus (HS) taken from Otto (1981).

Data suggest that Finnish populations of the nominate *A. g. gentilis* are slightly larger in their wing length and body mass than other Scandinavian populations (Tornberg et al., 2006), although the reasons for this remain unclear. It is possible that they represent a slightly more northern clinal population with bigger proportions. Alternatively, it may reflect a slightly more continental climate than Norway and Sweden (R. Tornberg, personal communication). In addition, we noticed that the Danish specimens included here were on average smaller than the other Scandinavian specimens, again possibly due to clinal variation. ANOVA tests were used to detect statistical differences between the modern populations for each country. The results show that Norway and Sweden did not differ; Denmark was often statistically different to Norway, Sweden and Finland; Norway and Sweden rarely differed from Finland (full ANOVA results in File S1). For these reasons, we have grouped Norway and Sweden together but kept Denmark and Finland separate when drawing comparisons.

### 2.2 Archaeological material

In general *A. gentilis* is not frequent within the archaeological record for Norway (Walker et al., 2019). Morphologically, the osteology of *A. gentilis* is not easily confused with any other species, and we are confident of specimen identification. Despite this all specimens were confirmed using the extensive modern comparative collections held at the University Museum of Bergen. In total 89 Medieval specimens were included within this study (Table 1). It is worth noting here that the 89 bones do not represent 89 individuals, although there is a possibility that some of these bones would have come from the same individual. The Medieval bones date to 1030–1537 Common Era (CE) and come from only eight sites, all from the urban contexts of Oslo, Bergen and Trondheim (Table 1). Most Medieval bones come from female individuals and are likely to be linked to the practice of falconry (Walker et al., 2019). Most of the archaeological bones were limb elements; this may be due to taphonomic bias as they are more robust than for example cranial remains. However, this limited the skeletal elements that could be used for comparison. Within this paper, we focus on the humerus, ulna, carpometacarpus, femur, tibiotarsus and tarsometatarsus.

**TABLE 1:** All Norwegian Medieval sites which contain the skeletal remains of *Accipiter gentilis*.

Site name	JS number	*A. gentilis* NISP	Location	Date range of site	Site references
Mindets Tomt	537	36	Oslo	Medieval (1025–1350 CE)	Archive Natural History, University Museum of Bergen (Lie, 1988)
Oslogate 7, Gamlebyen	599	2	Oslo	Medieval (1150–1600 CE)	Archive Natural History, University Museum of Bergen (Lie, 1979)
Nordre Felt I, Gamlebyen	809	1	Oslo	Medieval (1030–1537 CE)	Archive Natural History, University Museum of Bergen
Nordre Felt II, Gamlebyen	702	28	Oslo	Medieval (1030–1537 CE)	Archive Natural History, University Museum of Bergen
Bryggen	397,401, 529, 540	18	Bergen	Medieval (1030–1537 CE)	Archive Natural History, University Museum of Bergen
Dreggsalmenningen	630	1	Bergen	Medieval (1170–1527 CE)	Archive Natural History, University Museum of Bergen (Undheim, 1985, unpublished report)
Finnegården 3A	1237	1	Bergen	Medieval (1030–1537 CE)	Archive Natural History, University Museum of Bergen (Golembnik, 1993, unpublished report)
Erkebispegården	845	2	Trondheim	Medieval (Ca. 1250–1537 CE Phases 4–7)	Archive Natural History, University Museum of Bergen (Hufthammer, 1999)

*Note*: The JS number is a unique number given to each archaeological faunal assemblage within the University Museum of Bergen collections. NISP refers to the number of identified specimens of *A. gentilis*; we have only included the humerus, ulna, carpometacarpus, femur, tibiotarsus and tarsometatarsus in this figure.

### 2.3 Data analysis

**FIGURE 3:**
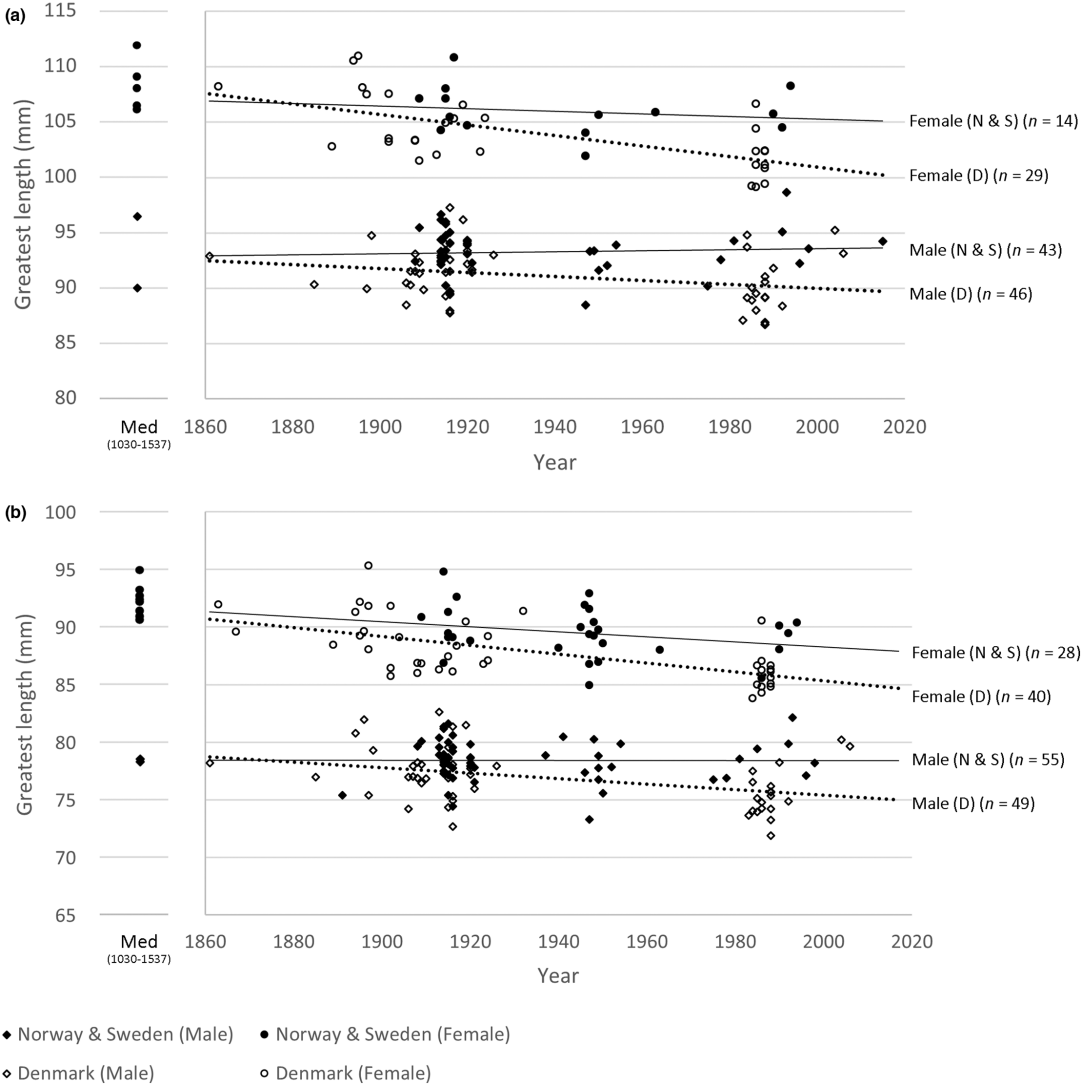
Greatest length of modern and archaeological *Accipiter gentilis gentilis*. (a) Humerus and (b) Femur. Norwegian Medieval specimens are plotted on the left‐hand side. The trendline is calculated as a linear regression (the equation and *R*‐squared value for each line are as follows: a, Female (N & S), *y* = −0.0118*x* + 128.28, *R* ^2^ = .029; Female (D), *y* = −0.0477*x* + 196.32, *R* ^2^ = .398; Male (N & S), *y* = 0.0046*x* + 84.378, *R* ^2^ = .0048; Male (D), *y* = −0.0179*x* + 125.74, *R* ^2^ = .0784. b, Female (N & S), *y* = −0.022*x* + 132.29, *R* ^2^ = .07; Female (D), *y* = −0.0385*x* + 162.38, *R* ^2^ = .3733; Male (N & S), *y* = −0.0005*x* + 79.359, *R* ^2^ = 6E‐05; Male (D), *y* = −0.024*x* + 123.38, *R* ^2^ = .1532).

We first explored differences in size between groups using descriptive statistics in PAST 4.03 (Hammer et al., 2001). All data were tested for normality by looking at the variances and the Shapiro–Wilk test for normality (see File S1). Principal Components Analysis (PCA) was used to establish which measurements were most responsible for the observed differences. Two separate PCAs were performed, one on the modern specimens only (including Norwegian, Swedish, Danish and Finnish modern specimens; File S2) and a second with both modern and archaeological specimens (including Norwegian, Swedish, Danish and Finnish modern and Norwegian Medieval specimens; File S4). To test for main and interaction effects of time, sex and country on greatest lengths (GL) of the humerus and femur of modern Northern Goshawks from Norway, Sweden and Denmark, we performed an Analysis of Covariance (ANCOVA) in R Statistical Software (v4.1.1; R Core Team, 2021), with the factor Time as the covariate and the factors Sex (2 levels), and Country (2 levels: Denmark and (Norway + Sweden) grouped together). For the humerus, *n* = 132, of which *n* = 57 for Norway and Sweden combined (43 males, 14 females), and *n* = 75 from Denmark (46 males, 29 females). For the femur, *n* = 172, with *n* = 83 for Norway and Sweden combined (55 males and 28 females) and *n* = 89 from Denmark (49 males, 40 females). Finnish modern specimens were excluded from the linear regression (Figure 3) and the ANCOVA, because of the 52 Finnish specimens available, only 7 predate the year 2000, preventing a detailed look into the past century.

To test for statistical differences between the mean greatest lengths of Norwegian Medieval specimens and modern Norwegian specimens of *A. g. gentilis*, we used a 10,000‐iteration Fisher's permutation test in R. The permutation test is somewhat similar to the bootstrap but differs from it in that a permutation test resamples without replacement. First, the sample means for each group and the difference between these means is computed. The data are then pooled and randomly permuted. The means and difference in mean for the permutated samples are computed. This process is then repeated *n* times for all possible permutations of the data, resulting in a frequency distribution of the mean difference. The 95% confidence interval and *p*‐values can then be calculated. We considered *p*‐values ≤ .05 statistically significant.

## 3 RESULTS

### 3.1 Modern Northern Goshawks

The results of the Principal Components Analysis (PCA) showed that the greatest length of the bone is the driving factor for size differences in limb elements of modern *A. g. gentilis* males and females (see File S2). We therefore present the results of the greatest length in detail within the paper (Table 2), with other measurements being presented in the File S3.

**TABLE 2:** The greatest length (GL) for the six limb elements analysed.

	Sex	No. specimens	Observed range (mm)	Mean ± standard error
Humerus GL				
*A. g. gentilis* (Norway & Sweden)	♂	44	88.49–98.67	93.28 ± 0.30
*A. g. gentilis* (Norway & Sweden)	♀	15	101.92–110.83	105.92 ± 0.55
*A. g. gentilis* (Denmark)	♂	46	86.73–97.28	91.03 ± 0.38
*A. g. gentilis* (Denmark)	♀	29	99.13–110.98	104.01 ± 0.59
*A. g. gentilis* (Finland)	♂	22	89.69–97.84	93.95 ± 0.48
*A. g. gentilis* (Finland)	♀	30	103.11–111.41	106.44 ± 0.33
*A. gentilis* (Medieval)	♂	2	90.00–96.46	93.23 ± 3.23
*A. gentilis* (Medieval)	♀	5	106.10–111.92	108.31 ± 1.05
Ulna GL				
*A. g. gentilis* (Norway & Sweden)	♂	21	98.60–109.40	103.94 ± 0.51
*A. g. gentilis* (Norway & Sweden)	♀	7	113.50–121.15	116.43 ± 1.03
*A. g. gentilis* (Denmark)	♂	23	96.48–106.94	100.95 ± 0.58
*A. g. gentilis* (Denmark)	♀	21	110.66–121.41	115.24 ± 0.73
*A. g. gentilis* (Finland)	♂	22	98.97–108.73	104.90 ± 0.51
*A. g. gentilis* (Finland)	♀	30	112.61–121.94	117.49 ± 0.38
*A. gentilis* (Medieval)	♂	3	102.55–103.05	102.83 ± 0.15
*A. gentilis* (Medieval)	♀	4	117.89–122.04	120.23 ± 0.88
Carpometacarpus GL				
*A. g. gentilis* (Norway & Sweden)	♂	21	53.93–60.97	57.30 ± 0.33
*A. g. gentilis* (Norway & Sweden)	♀	8	62.56–67.11	63.92 ± 0.52
*A. g. gentilis* (Denmark)	♂	26	52.81–58.94	55.37 ± 0.31
*A. g. gentilis* (Denmark)	♀	19	60.60–67.21	63.16 ± 0.45
*A. g. gentilis* (Finland)	♂	22	55.62–60.59	57.83 ± 0.28
*A. g. gentilis* (Finland)	♀	30	62.22–67.10	65.22 ± 0.23
*A. gentilis* (Medieval)	♂	2	56.21–57.99	57.10 ± 0.89
*A. gentilis* (Medieval)	♀	3	64.71–67.48	65.97 ± 0.81
Femur GL				
*A. g. gentilis* (Norway & Sweden)	♂	57	73.30–82.13	78.40 ± 0.23
*A. g. gentilis* (Norway & Sweden)	♀	29	84.93–94.78	89.41 ± 0.40
*A. g. gentilis* (Denmark)	♂	49	71.88–82.63	76.83 ± 0.36
*A. g. gentilis* (Denmark)	♀	40	83.81–95.30	87.91 ± 0.42
*A. g. gentilis* (Finland)	♂	22	75.48–82.35	78.81 ± 0.38
*A. g. gentilis* (Finland)	♀	30	86.31–92.95	89.68 ± 0.30
*A. gentilis* (Medieval)	♂	2	78.27–78.50	78.39 ± 0.12
*A. gentilis* (Medieval)	♀	8	90.60–94.89	92.26 ± 0.49
Tibiotarsus GL				
*A. g. gentilis* (Norway & Sweden)	♂	23	97.48–107.22	103.42 ± 0.46
*A. g. gentilis* (Norway & Sweden)	♀	9	113.13–121.22	116.14 ± 0.86
*A. g. gentilis* (Denmark)	♂	25	96.70–106.07	101.31 ± 0.48
*A. g. gentilis* (Denmark)	♀	23	111.63–122.31	116.29 ± 0.62
*A. g. gentilis* (Finland)	♂	22	98.34–107.41	103.89 ± 0.49
*A. g. gentilis* (Finland)	♀	30	113.15–120.94	117.69 ± 0.36
*A. gentilis* (Medieval)	♂	0	–	–
*A. gentilis* (Medieval)	♀	10	115.29–123.28	119.72 ± 0.81
Tarsometatarsus GL				
*A. g. gentilis* (Norway & Sweden)	♂	21	71.91–79.06	76.48 ± 0.46
*A. g. gentilis* (Norway & Sweden)	♀	10	82.50–88.34	84.42 ± 0.60
*A. g. gentilis* (Denmark)	♂	25	70.37–79.32	74.95 ± 0.43
*A. g. gentilis* (Denmark)	♀	21	81.22–89.02	84.57 ± 0.47
*A. g. gentilis* (Finland)	♂	22	73.13–80.37	77.27 ± 0.39
*A. g. gentilis* (Finland)	♀	30	81.63–89.92	86.29 ± 0.31
*A. gentilis* (Medieval)	♂	3	73.08–79.01	75.20 ± 1.91
*A. gentilis* (Medieval)	♀	8	84.43–89.50	87.12 ± 0.57

*Note*: The *A. g. gentilis* data are made up of modern specimens from Norway, Sweden and Denmark. The *A. g. gentilis* (Finland) data represent only modern specimens from Finland (not including Lapland). The *A. gentilis* (Medieval) are archaeological specimens from Medieval Norwegian contexts. All the measurements are in mm.

Humerus and femur greatest lengths of modern specimens of Northern Goshawks were plotted onto Figure 2. The contemporary data of the specimens in Figure 3 date from 1861 to 2015 for males, and between 1863 and 1994 for females. An ANCOVA showed that there are significant effects of Year, Sex and Country, as well as significant interactions between Year and Sex and between Year and Country, on the humerus greatest length. For the femur greatest length, an ANCOVA showed a significant effect of Year, Sex and Country, as well as significant interactions between Year and Sex and between Year and Country (Table 3). These results demonstrate that Norwegian and Swedish contemporary males have not changed in size over the past century (Figure 3a,b). However, females show a decrease in greatest length of 2%–3% over the past century (Figure 3a,b). The modern Danish specimens showed a decrease in the size of both males and females; the males decreased by around 3%–4% and the females by 6%–7% (Figure 3a,b). In both the humerus (Figure 3a) and the femur (Figure 3b), the decrease in size over the past century has been more pronounced within females. The ulna, carpometacarpus, tibiotarsus and tarsometatarsus show a similar pattern to the humerus (Figure 3a) and femur (Figure 3b) but are not presented here.

**TABLE 3:** Results of Analysis of Covariance (ANCOVA) for the humerus and femur of modern *Accipiter gentilis gentilis* specimens from Norway, Sweden and Denmark for the factors Year, Sex (2 levels), Country (2 levels) and interactions between these.

	df	Sum sq	Mean sq	*F*‐Value	*p*‐Value
Humerus (*n* = 132)					
Year	1	102.4	102.4	18.8424	2.886e‐05***
Sex	1	4535.8	4535.8	834.2298	<2.2e‐16***
Country	1	138.1	138.1	25.3966	1.582e‐06***
Year:Sex	1	36.7	36.7	6.7527	.01048*
Year:Country	1	25.1	25.1	4.6086	.03373*
Residuals	126	685.1	5.4		
Femur (*n* = 172)					
Year	1	80.9	80.9	18.7727	2.543e‐05***
Sex	1	4879.7	4879.7	1132.7669	<2.2e‐16***
Country	1	109.2	109.2	25.3403	1.241e‐06***
Year:Sex	1	20.7	20.7	4.8025	.02981*
Year:Country	1	23.8	23.8	5.5158	.02002*
Residuals	166	715.1	4.3		

*Note*: Data for Norway and Sweden are combined, data for Finland are excluded from the analysis (see text for explanation). For the humerus, *n* = 132, of which *n* = 57 for Norway & Sweden combined (43 males, 14 females), and *n* = 75 from Denmark (46 males, 29 females). For the femur, *n* = 172, with *n* = 83 for Norway & Sweden combined (55 males and 28 females) and *n* = 89 from Denmark (49 males, 40 females). Significance codes: 0 ‘***’ .001 ‘*’ .05 ‘.’ .1 ‘ ’ 1.

**FIGURE 4:**
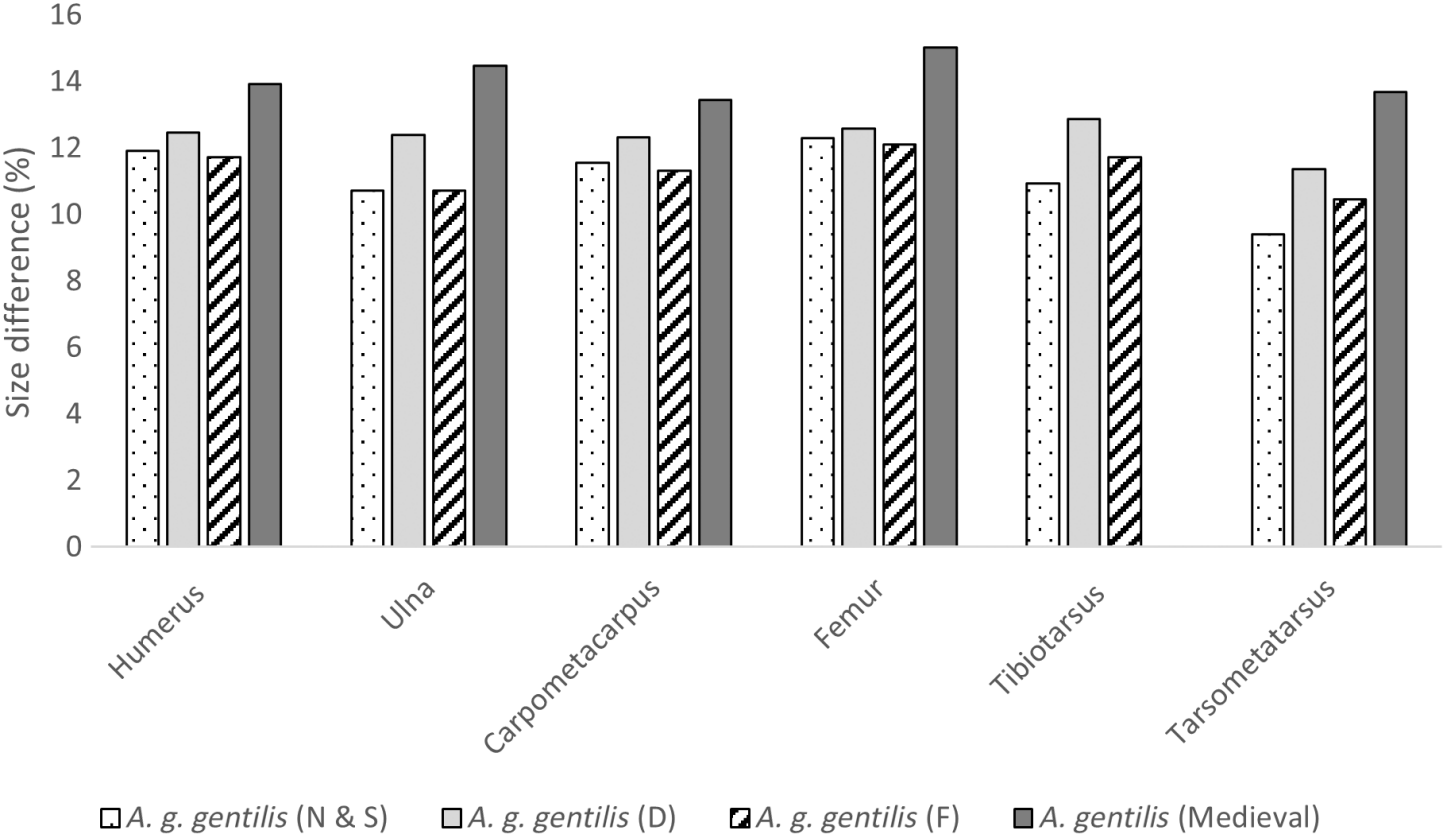
Percentage size difference between male and female *Accipiter gentilis gentilis*. Comparing sexual dimorphism of modern populations and the Medieval Norwegian populations. The percentage size difference was calculated based on the mean greatest length of males and females; mean figures can be found in Table 2. Unfortunately, there are no male *A. gentilis* tibiotarsi within the Norwegian Medieval material, as a result it is left blank.

Sexual dimorphism of the modern populations examined here was calculated as the percentage difference between the mean female greatest length and the mean male greatest length for each element (Figure 4). The results showed that the percentage difference varied by element, but females were on average between 10% and 12% larger than males.

### 3.2 Archaeological Northern Goshawk

The PCAs comparing contemporary and archaeological populations show that the greatest length of the bone elements accounts for most of the variation (see File S4). Norwegian Medieval specimens were included in Figure 3 and appear to show little change in males from the Medieval period to modern specimens. The Medieval females appear larger on average than their modern counterparts (Table 2).

**FIGURE 5:**
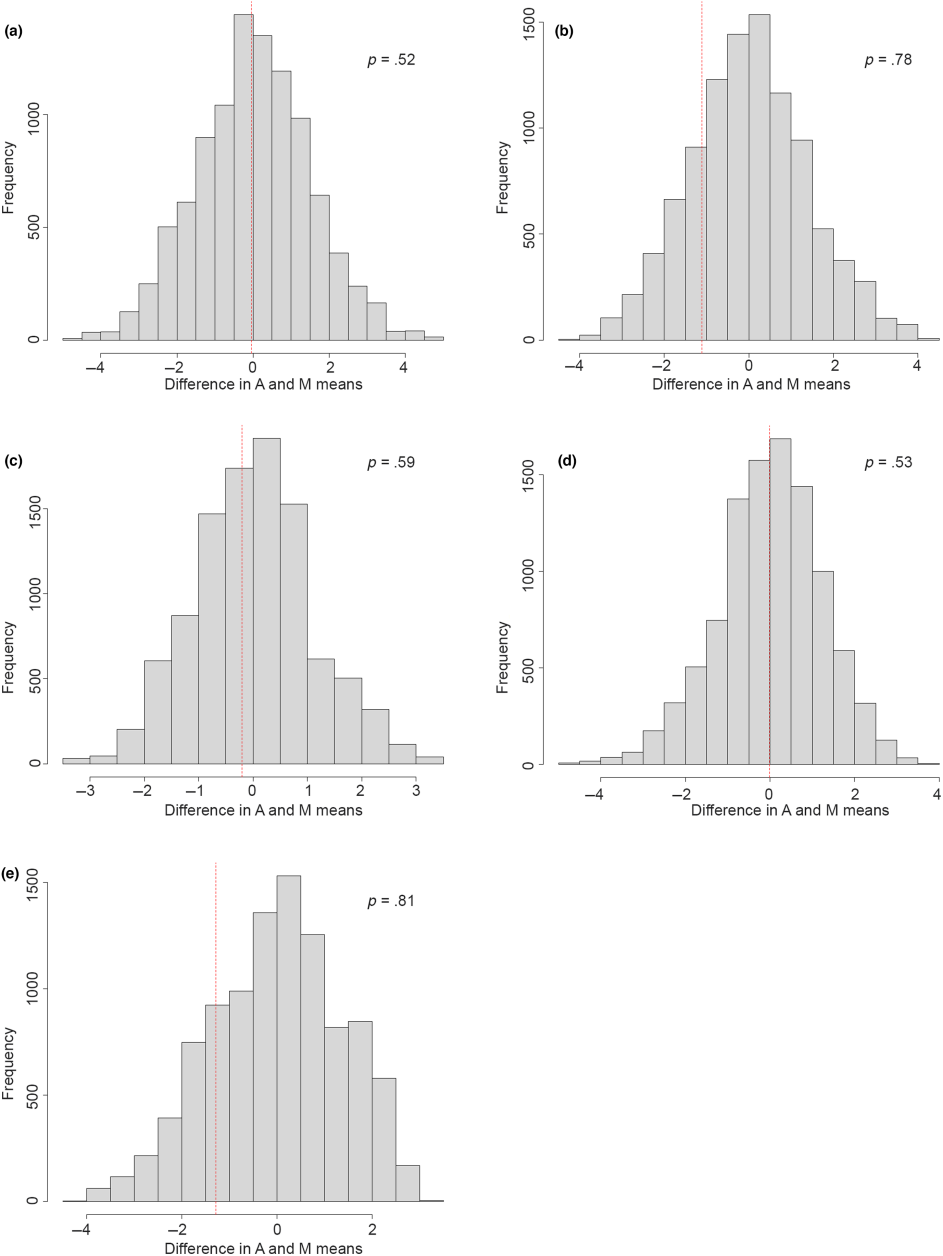
Frequency distributions of 10,000 permutations of the difference in mean greatest length of the limb elements between the modern male specimens (M) and the Medieval specimens (A) from Norway. The red dotted line indicates the observed difference between the modern and Medieval specimens. (a) Humerus (modern *n* = 44, Medieval *n* = 2), (b) ulna (modern *n* = 21, Medieval *n* = 3), (c) carpometacarpus (modern *n* = 21, Medieval *n* = 2), (d) femur (modern *n* = 57, Medieval *n* = 2), (e) tarsometatarsus (modern *n* = 21, Medieval *n* = 3).

**FIGURE 6:**
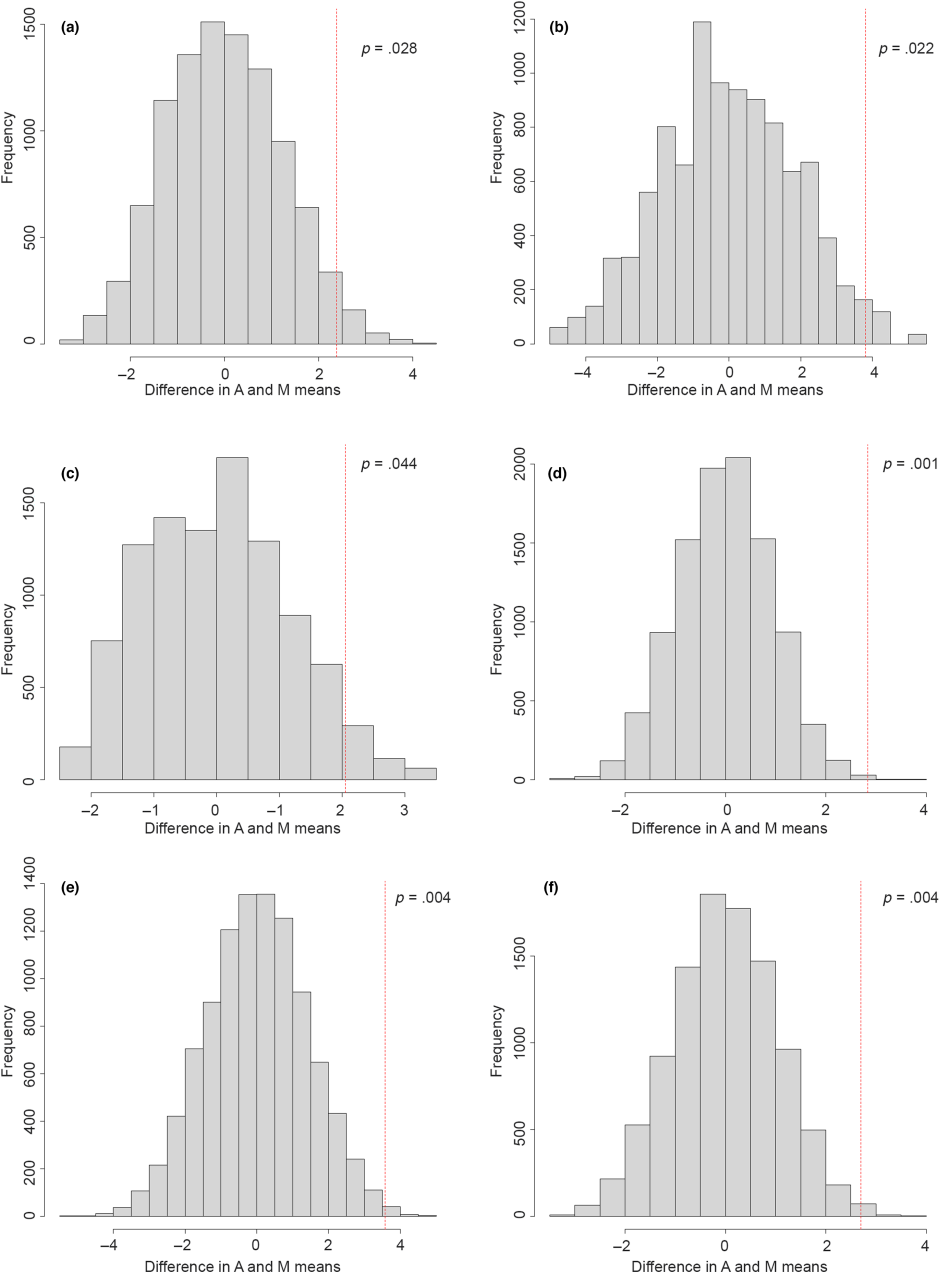
Frequency distributions of 10,000 permutations of the difference in mean greatest length of the limb elements between the modern female specimens (M) and the Medieval specimens (A) from Norway. The red dotted line indicates the observed difference between the modern and archaeological specimens. (a) Humerus (modern *n* = 15, Medieval *n* = 5), (b) ulna (modern *n* = 7, Medieval *n* = 4), (c) carpometacarpus (modern *n* = 8, Medieval *n* = 3), (d) femur (modern *n* = 37, Medieval *n* = 8), (e) tibiotarsus (modern *n* = 9, Medieval *n* = 10), (f) tarsometatarsus (modern *n* = 10, Medieval *n* = 8).

Fisher's permutation tests show that for male Northern Goshawks, the observed difference between the archaeological specimens (A) and modern (M) male specimens fell within the 95% confidence interval of the randomised permutations for all five elements available (humerus, ulna, carpometacarpus, femur and tarsometatarsus; see Figure 5). This indicates that there is no difference between the archaeological male specimens and modern male Goshawks. In contrast, for female Goshawks, the observed difference fell consistently outside the 95% confidence interval of the randomised permutations for all six skeletal elements (humerus, ulna, carpometacarpus, femur, tibiotarsus and tarsometatarsus; see Figure 6), indicating a significant difference between Medieval and contemporary females. The percentage difference between male and female Medieval Goshawks varies depending on the skeletal element, but females are between 13.5% and 15% larger than males in the material from the Medieval period (Figure 4).

## 4 DISCUSSION

### 4.1 Changes to modern Northern Goshawk populations

The results presented here represent the first detailed metric analysis of modern and archaeological skeletal elements of *A. g. gentilis* and provide important modern comparative measurements for those studying Northern Goshawks in Scandinavia (Table 2; File S3). The works of Otto (1981) and Schmidt‐Burger (1982) present size ranges for Northern Goshawk from central Europe, but only a limited number of the larger Scandinavian specimens had been included. The ranges presented here are therefore more accurate for comparison with specimens in Northern Europe.

Our results show a steady decline in length of the skeletal elements over the past century, particularly in females (Table 3; Figure 3). This decline is likely associated with an overall decrease in body size from at least 1900 onwards. This shows that a body size decrease, as seen in Finland (Tornberg et al., 1999, 2014) and Denmark (Yom‐Tov & Yom‐Tov, 2006), also occurred in Norway and Sweden. However, the Norwegian and Swedish male specimens appear to show little change over the past century (Figure 2), whilst those from Denmark and Finland (Tornberg et al., 1999) do show a decrease in size. The decrease seen in Norwegian and Swedish material shows that females appear to have declined on average 2%–3% whilst male size has remained fairly stable over the past century. The Danish specimens show a decrease of 6%–7% in females and 3%–4% in males. The larger decrease in females matches the results from Yom‐Tov and Yom‐Tov (2006) who also noted a greater decline in female body size. There is a lack of data for Danish Goshawks around 1940–1980 (Figure 3), and it appears that at some point during this period the decline in size occurs, as specimens prior to the 1940s are of a similar size to those found in Norway and Sweden (Figure 3). Overall, it therefore appears that female Northern Goshawks within the Nordic countries are collectively decreasing in size.

The spatiotemporal variation in reversed sexual size dimorphism (RSD) in Goshawks is interesting from an evolutionary perspective. Most birds of prey show RSD (Székely et al., 2007). Adaptive reasons for the variation in this trait have been debated for more than a century (Schoenjahn et al., 2020), and Krüger (2005) identified more than 20 explanatory hypotheses involving both sexual and natural selection. For instance, sexual selection could favour smaller males if this makes them more agile in aerobatic territorial displays (Widén, 1984). Alternatively, reversed SSD may result from natural selection, where larger females are selected for due to their better abilities to protect the nest and offspring against predators (Schoenjahn et al., 2020). A third possibility is that smaller body size is selected for in males to improve their hunting abilities because of their special role of bringing food to both chicks in the nest and the attending female (Andersson & Nordberg, 1981; Krüger, 2005). In support of the latter hypothesis, the degree of RSD in raptors increases with the proportion of agile prey (such as birds) in the diet (Andersson & Nordberg, 1981).

If the availability of potential prey species of a raptor changes over time, this might affect the evolution of body size in both sexes and perhaps explain the temporal changes in RSD observed in Scandinavian Goshawks (Tornberg et al., 1999, 2006; Yom‐Tov & Yom‐Tov, 2006; this study). There are several possible reasons for the patterns we are seeing in the contemporary Northern Goshawk populations. The Goshawks' diet for northern Europe consists predominantly of forest grouse species (*Tetrao urogallus*, *Tetrao tetrix*, *Bonasa bonasia* and *Lagopus lagopus*; Höglund, 1964; Huhtala, 1976; Lindén & Wikman, 1983; Selås, 1989; Tornberg, 1997; Widén, 1987). However, with declines in forest grouse populations (Gregersen & Gregersen, 2009; Selås et al., 2011; Storch, 2007), Northern Goshawks have shifted their focus to smaller, more available prey, such as corvids, thrushes and pigeons (Selås, 1989; Tornberg et al., 2014; Tornberg & Colpaert, 2001; Widén, 1987). In Norway and Sweden, Wood Pigeon (*Columba palumbus*) have increased in numbers and range over the past 50 years (Gjershaug et al., 1994; Haftorn, 1971; Ottosson et al., 2012) and have become a key contributor to the Goshawk diet (Johansen et al., 2007; Verdal & Selås, 2010). However, the smaller thrush species by far contribute the most to *A. gentilis* diets in Norway (Johansen et al., 2007; Verdal & Selås, 2010). It has been this dietary change to smaller prey which has led some to believe this is the cause of decline in size of Finnish and Danish *A. g. gentilis* populations in the 20th century (Tornberg et al., 1999, 2006; Yom‐Tov & Yom‐Tov, 2006). It is possible that we are seeing a similar pattern in Norway and Sweden, whereby body sizes of females are decreasing to become more adept and agile at catching smaller prey. In central and western Europe, Northern Goshawks have long adapted to hunting in smaller wooded areas, parks and farmland, and pigeons and corvids are highly represented in the diet from these areas (Rutz et al., 2006). As a result of this smaller prey, the body size of *A. gentilis* from central and western Europe is generally smaller than seen in Scandinavia.

The evidence for selective smaller body size due to dietary changes is compelling. In addition, Northern Goshawks display a clinal body size pattern, whereby populations in northern and eastern Europe are larger than those in western and central Europe (Cramp & Simmons, 1980). This pattern is visible in the modern specimens analysed within this paper as well; Danish populations seem slightly smaller and Finnish specimens slightly larger on average. Bergmann's rule is often used to explain such clinal differences. Bergmann's rule is based upon thermoregulation and suggests that individuals in the colder north are larger than individuals in the warmer south (Bergmann, 1847). A review of Bergmann's rule has found that 72% of the 94 bird species tested do conform to the rule, suggesting that Bergmann's rule is a valid ecological generalisation for bird species (Meiri & Dayan, 2003). In line with this, Sunde (2002) has suggested that the clinal size difference seen in *A. gentilis* may be a selective trait to guard against starvation in the harsher climates of the north, and to take advantage of larger prey. This theory is known as the starvation resistance hypothesis and suggests clinal size variation is attributed to availability of food (Blackburn et al., 1999). Over the past 100 years global surface temperatures have increased by 1°C (Collins et al., 2013), having great implications on seasonal temperatures, increased precipitation and reduced snow cover, and leading to changes in habitat for many taxa. It is possible that *A. g. gentilis* is decreasing in body size as a response to warmer climates, which are amplified at higher latitudes (Pithan & Mauritsen, 2014). It should be noted that dietary and climatic changes are not mutually exclusive. Changes in climate and reduction of habitat impact the prey available and as a result species alter their diet. It appears highly likely that the decline is a response to a change in diet and/or climate. If the decline in size was related to climate, it is possible that the already small males would be less affected, with greater selection pressure on the larger females in a warming climate. This would also be the case if body size is declining as an adaptation to the capture of smaller prey; males that are already a smaller size would be less affected than the larger females. The slightly different responses seen across Scandinavia possibly reflects the changing habitats and diets within local populations.

### 4.2 Archaeological Northern Goshawk from Norway

Medieval female *A. g. gentilis* are on average 3%–4% larger than contemporary females (Table 2). There were far fewer Medieval male specimens for Norway, and on average they were slightly larger than modern males (Table 2). As a result, sexual dimorphism in Medieval *A. g. gentilis* was higher than seen today (Figure 3). Sexual dimorphism in the skeletal elements of *A. g. gentilis* of modern populations is between 10% and 12% whilst in the Norwegian Medieval populations it was between 13.5% and 15%.

The contemporary evidence suggests females are decreasing in relation to smaller prey, which would suggest that the larger Medieval females had access to bigger prey. Greater forested areas would have meant more forest grouse species and more habitat for the Goshawks. Archaeological evidence across Europe would suggest that *A. g. gentilis* were more abundant in the past (Heinrich, 1998). This is based on a comparison within the archaeological record with the now more dominant Common Buzzard (*Buteo buteo*). However, Northern Goshawk are more prevalent in archaeological assemblages (Heinrich, 1998). Newly established agricultural pastures close to forested areas have strongly favoured Northern Goshawk. But, as more areas have been cleared and forested areas have been heavily exploited, we now see a dominance of the Common Buzzard over the Northern Goshawk (Heinrich, 1998). The reduction of forested areas has led to a decline in Northern Goshawk numbers in Norway (Grønlien, 2004; Selås et al., 2008). It would seem likely that Northern Goshawk in the Medieval period were larger based on bigger prey items and habitat availability. The fact that we see little change in males from the Medieval period to now may be that their dependence on smaller prey has not been as heavily affected as the females' dependence on larger prey species.

There are certain caveats that should be highlighted here. The Medieval Northern Goshawks are all likely to represent falconry birds (Walker et al., 2019). These are birds that are taken from the wild and trained for the sport of hunting. It is possible that only the largest and strongest birds were used in this practice which would distort our view of the natural population. Birds of prey from Scandinavia were highly valuable and traded across Europe during this period (Lie, 2018). Although larger birds likely demanded a higher price, many smaller birds would have been taken from the wild as well. A further point of discussion is whether the larger size of the Medieval Goshawks could be explained by them belonging to the larger subspecies *A. g. buteoides*. The location of Medieval finds is not within the current distribution of *A. g. buteoides*, but it is unclear whether this subspecies would have had the same or a larger distribution in the past. However, if our specimens did represent this subspecies, we would have expected the Medieval male Goshawks also to have been larger, and this was not the case. Finally, the relatively small sample size should also be taken into consideration. How much the specimens reported here reflect past populations is difficult to say, but given the geographic and temporal spread of the remains we believe they represent a genuine size difference between modern populations.

The only other archaeological Northern Goshawk specimens we have from Norway are two female individuals from the Viking Age Gokstad burials. These do not seem to fit with the Medieval pattern of being bigger than their modern‐day counterparts. Both females are around the average size of modern populations (File S5). It is difficult to draw too many conclusions from these as they represent only two birds. The Gokstad specimens represent the first evidence of falconry from Norway, dating between 895 and 905 CE (Bill & Daly, 2012; Christensen et al., 1992; Nicolaysen, 1882). As such it is entirely possible that these two individuals derived from somewhere other than Norway and were imported from abroad. Given the wealth of exotic items from across Europe within the burial (Pedersen et al., 2016), the two Goshawks could be from Britain, France, Denmark or other parts of central Europe. More Northern Goshawk specimens from the Viking Age would allow for a more complete comparison with the Medieval and contemporary populations.

## 5 CONCLUSION

Our study shows how Northern Goshawks in Scandinavia have declined in body size during the last century, and are smaller than their Medieval counterparts, particularly the females. The decline in goshawk body size appears to be linked to a decline in forest habitats due to deforestation and a concomitant shift towards smaller prey which resulted in smaller body size in Northern Goshawks. Our data highlight how quickly body size changes can occur in birds as a result of environmental factors. Moreover, the study also highlights the importance of modern reference material from across geographical and temporal scales held within museum collections (see also Lister, 2011). Such material provides insights into changes in species over the past century and helps us understand how species might respond to future environmental change.

## AUTHOR CONTRIBUTIONS

**Samuel J. Walker:** Conceptualization (equal); data curation (equal); formal analysis (equal); funding acquisition (lead); investigation (lead); methodology (equal); project administration (equal); validation (equal); visualization (equal); writing – original draft (lead); writing – review and editing (equal). **Terje Lislevand:** Methodology (equal); resources (equal); supervision (supporting); validation (equal); visualization (equal); writing – review and editing (equal). **Hanneke J. M. Meijer:** Conceptualization (equal); data curation (equal); formal analysis (equal); investigation (equal); methodology (equal); resources (equal); supervision (lead); validation (equal); visualization (equal); writing – original draft (equal).

## FUNDING INFORMATION

This paper resulted from SJW's PhD project funded by the University Museum of Bergen. Additional funding was provided by two SYNTHESYS grants (DK‐TAF‐2419 and FI‐TAF‐2548) which allowed SJW to measure *Accipiter gentilis gentilis* specimens from the Natural History Museum of Denmark and the Finnish Museum of Natural History. No other funding agencies in the public, commercial, or not‐for‐profit sectors were involved.

## CONFLICT OF INTEREST STATEMENT

The authors have no competing interests to declare.

### OPEN RESEARCH BADGES

This article has earned an Open Data badge for making publicly available the digitally‐shareable data necessary to reproduce the reported results. The data is available at https://datadryad.org/stash/share/NZXdnpJtNWz_G6wo6As9PhDpYzT9fYZFglA_EqeOn_g.

## Supporting information


File S1.

File S2.

File S3.

File S4.

File S5.

## Data Availability

The data that support the findings of this study are available within the paper and the Supporting Information of this article. In addition, the raw data for this article can be found on DataDryad: https://datadryad.org/stash/share/NZXdnpJtNWz_G6wo6As9PhDpYzT9fYZFglA_EqeOn_g.

## APPENDIX 1 LIST OF MODERN *ACCIPITER GENTILIS GENTILIS* SPECIMENS USED FOR COMPARISON.

### 1.1

University Museum of Bergen, Bergen, (prefix B and BM); B 1, B 313, B 414, B 1240, B 3093, B 3145, B 3189, B 3577, B 4389, B 4390, B 4391, B 4393, B 4394, B 4415, B 4459, B 4460, B 4461, B 4462, B 4463, B 4464, B 4466, B 4467, B 4468, B 4469, B 4470, B 4471, B 4472, B 4473, B 4474, B 4475, B 4476, B 4477, B 5460, B 5461, B 7407, B 7410, B 7425, B 7428, B 7763, B 9014, B 9016, B 9018, B 9487, B 9610, B 9613, B 9798, B 9885, B 10493, BM 7764, BM 7765, BM 8071, BM 8072, BM 8073, BM 8175, BM 8293, BM 9355, BM 9476, BM 10053, BM 10193, BM 10199 BM 10212, BM 10235, BM 10494, BM 10495, BM 10541, BM 10673, BM 10700, BM 10707, BM 10727, BM 10731, BM 10766, BM 10778, BM 10779, BM 10784, BM 10799, BM 10806, BM 10851.

Natural History Museum of Denmark, Copenhagen (prefix NHMD); NHMD 125213, NHMD 125230, NHMD 306501, NHMD 306502, NHMD 306504, NHMD 306505, NHMD 306506, NHMD 306507, NHMD 306509, NHMD 306511, NHMD 306512, NHMD 306513, NHMD 306514, NHMD 306515, NHMD 306516, NHMD 306517, NHMD 306519, NHMD 306520, NHMD 306521, NHMD 306523, NHMD 306524, NHMD 306525, NHMD 306526, NHMD 306527, NHMD 306529, NHMD 306530, NHMD 306531, NHMD 306536, NHMD 306537, NHMD 306539, NHMD 306549, NHMD 306550, NHMD 306551, NHMD 306552, NHMD 306553, NHMD 306554, NHMD 306555, NHMD 306556, NHMD 306557, NHMD 306558, NHMD 306559, NHMD 306560, NHMD 306564, NHMD 306565, NHMD 306568, NHMD 306570, NHMD 306571, NHMD 306573, NHMD 306574, NHMD 306575, NHMD 306577, NHMD 306578, NHMD 306579, NHMD 306580, NHMD 306582, NHMD 306585, NHMD 306587, NHMD 306590, NHMD 306591, NHMD 306592, NHMD 306593, NHMD 306594, NHMD 306595, NHMD 306596, NHMD 306597, NHMD 306599, NHMD 306602, NHMD 306603, NHMD 306605, NHMD 306606, NHMD 306607, NHMD 306608, NHMD 306610, NHMD 306611, NHMD 306612, NHMD 306617, NHMD 306619, NHMD 306620, NHMD 306621, NHMD 306627, NHMD 306635, NHMD 306642, NHMD 306643, NHMD 306645, NHMD 306646, NHMD 306649, NHMD 306650, NHMD 306651, NHMD 306652, NHMD 306654, NHMD 306655, NHMD 306656, NHMD 306659, NHMD 306661, NHMD 306662, NHMD 306663, NHMD 306664, NHMD 306665, NHMD 306666, NHMD 306667, NHMD 306668, NHMD 306669, NHMD 306670, NHMD 306671, NHMD 306672, NHMD 306673, NHMD 306674, NHMD 306675, NHMD 306676, NHMD 306677, NHMD 306678.

Finnish Museum of Natural History, Helsinki (prefix KL); KL 25163, KL 25306, KL 25307, KL 25309, KL 25310, KL 25313, KL 25320, KL 25494, KL 25497, KL 25500, KL 25502, KL 25503, KL 25512, KL 26622, KL 26634, KL 26690, KL 26714, KL 26756, KL 26784, KL 26790, KL 26794, KL 26798, KL 26817, KL 26834, KL 26885, KL 26911, KL 27231, KL 27285, KL 27296, KL 27324, KL 27420, KL 27458, KL 27459, KL 27465, KL 27486, KL 27502, KL 27571, KL 27647, KL 27748, KL 27851, KL 27863, KL 27912, KL 27941, KL 30254, KL 31276, KL 31303, KL 31321, KL 31322, KL 31677, KL 31805, KL 31839, KL 32051.
