# Supplementary material for: A long‐term study of size variation in Northern Goshawk Accipiter gentilis across Scandinavia, with a focus on Norway
Source: Ecol Evol. 2023 Dec 7;13(12):e10789. doi: 10.1002/ece3.10789 (PMC10701624; doi:10.1002/ece3.10789)
Supplement: Supplementary file 1 — File S1. [file ECE3-13-e10789-s001.docx]

**Supporting Information File 1 (SIF1)**. ANOVA results.

# Female humerus Greatest Length (GL)

Female humerus GL shows the groups are normally distributed (Shapiro-Wilk p = 0.180). The Levene’s test for homogeneity of variance reports a p value of 0.007, this means that the variances are unequal, this compromises the assumptions of a one-way ANOVA. As a result, Welch’s F test (in the case of unequal variances) was consulted and included in Table 1 along with the one-way ANOVA results, as the p value of the Welch’s test was significant (p=0.02) it was possible to continue to Tukey’s pairwise (Table 2). Table 1 presents the results of the one-way ANOVA in standard form and Table 2 shows the Tukey’s pairwise results.

| **Table 1**, Female humerus GL one-way ANOVA | | | | | |
| --- | --- | --- | --- | --- | --- |
|  | **Sum of sqrs** | **df** | **Mean square** | ***F*** | ***p*(same)** |
| **Between groups** | 105.685 | 3 | 35.2284 | 5.679 | 0.001537 |
| **Within groups** | 434.223 | 70 | 6.20319 |  |  |
| **Welch *F* test** |  | 15.40 |  | 4.749 | 0.01553 |

One-way ANOVA results for the greatest length (GL) of the female humerus. Taking into account only modern material.

| **Table 2**, Female humerus GL ANOVA – Tukey’s pairwise | | | |  |  |  |
| --- | --- | --- | --- | --- | --- | --- |
|  | **Norway** | **Sweden** | **Denmark** | **Finland** |  |  |
| **Norway** | - | 0.479 | 0.515 | 0.574 |  |  |
| **Sweden** | 2.037 | - | 0.045* | 0.912 |  |  |
| **Denmark** | 1.955 | 3.785 | - | 0.002* |  |  |
| **Finland** | 1.820 | 0.934 | 5.305 | - |  |  |

# Male humerus Greatest Length (GL)

Male humerus GL shows the groups are normally distributed (Shapiro-Wilk p = 0.823). The Levene’s test for homogeneity of variance reports a p value of 0.164, so there is no significant difference. This means the assumptions of the ANOVA are safely met. Table 3 presents the results of the one-way ANOVA in standard form and Table 4 shows the Tukey’s pairwise results.

| **Table 3**, Male humerus GL one-way ANOVA | | | | | |
| --- | --- | --- | --- | --- | --- |
|  | **Sum of sqrs** | **df** | **Mean square** | ***F*** | ***p*(same)** |
| **Between groups** | 172.097 | 3 | 57.3657 | 10.70 | 3.244E-06 |
| **Within groups** | 578.963 | 108 | 5.36076 |  |  |

One-way ANOVA results for the greatest length (GL) of the male humerus. Taking into account only modern material.

| **Table 4**, Male humerus GL ANOVA – Tukey’s pairwise | | | |  |  |  |
| --- | --- | --- | --- | --- | --- | --- |
|  | **Norway** | **Sweden** | **Denmark** | **Finland** |  |  |
| **Norway** | - | 0.995 | 0.001* | 0.820 |  |  |
| **Sweden** | 0.335 | - | 0.004* | 0.713 |  |  |
| **Denmark** | 5.630 | 4.914 | - | >.001* |  |  |
| **Finland** | 1.230 | 1.503 | 6.870 | - |  |  |

# Female ulna Greatest Length (GL)

Female ulna GL shows the groups are normally distributed (Shapiro-Wilk p = 0.140). The Levene’s test for homogeneity of variance reports a p value of 0.032, this means that the variances are unequal, this compromises the assumptions of a one-way ANOVA. As a result, Welch’s F test (in the case of unequal variances) was consulted and included in Table 5 along with the one-way ANOVA results, as the p value of the Welch’s test was significant (p=0.05) it was possible to continue to Tukey’s pairwise (Table 6). Table 5 presents the results of the one-way ANOVA in standard form and Table 6 shows the Tukey’s pairwise results.

| **Table 5**, Female ulna GL one-way ANOVA | | | | | |
| --- | --- | --- | --- | --- | --- |
|  | **Sum of sqrs** | **df** | **Mean square** | ***F*** | ***p*(same)** |
| **Between groups** | 62.5686 | 2 | 31.2843 | 4.348 | 0.01766 |
| **Within groups** | 395.726 | 55 | 7.19501 |  |  |
| **Welch *F* test** |  | 15.6 |  | 3.709 | 0.04812 |

One-way ANOVA results for the greatest length (GL) of the female ulna. Taking into account only modern material.

| **Table 6**, Female ulna GL ANOVA – Tukey’s pairwise. No Swedish female ulna data was available. | | | | |
| --- | --- | --- | --- | --- |
|  | **Norway** | **Denmark** | **Finland** |  |
| **Norway** | - | 0.572 | 0.616 |  |
| **Denmark** | 1.434 | - | 0.013* |  |
| **Finland** | 1.334 | 4.167 | - |  |

# Male ulna Greatest Length (GL)

Male ulna GL shows the groups are normally distributed (Shapiro-Wilk p = 0.946). The Levene’s test for homogeneity of variance reports a p value of 0.941, this means that the variances are equal, and the assumptions of a one-way ANOVA are met. Table 7 presents the results of the one-way ANOVA in standard form and Table 8 shows the Tukey’s pairwise results.

| **Table 7**, Male ulna GL one-way ANOVA | | | | | |
| --- | --- | --- | --- | --- | --- |
|  | **Sum of sqrs** | **df** | **Mean square** | ***F*** | ***p*(same)** |
| **Between groups** | 192.311 | 2 | 96.1556 | 14.91 | 5.158E-06 |
| **Within groups** | 399.79 | 62 | 6.44823 |  |  |

One-way ANOVA results for the greatest length (GL) of the male ulna. Taking into account only modern material.

| **Table 8**, Male ulna GL ANOVA – Tukey’s pairwise. No Swedish male ulna data was available. | | | | |
| --- | --- | --- | --- | --- |
|  | **Norway** | **Denmark** | **Finland** |  |
| **Norway** | - | >.001* | 0.473 |  |
| **Denmark** | 1.434 | - | >.001* |  |
| **Finland** | 1.334 | 4.167 | - |  |

# Female carpometacarpus Greatest Length (GL)

Female carpometacarpus GL shows the groups are normally distributed (Shapiro-Wilk p = 0.324). The Levene’s test for homogeneity of variance reports a p value of 0.130, this means that the variances are equal, and the assumptions of a one-way ANOVA are met. Table 9 presents the results of the one-way ANOVA in standard form and Table 10 shows the Tukey’s pairwise results.

| **Table 9**, Female carpometacarpus GL one-way ANOVA | | | | | |
| --- | --- | --- | --- | --- | --- |
|  | **Sum of sqrs** | **df** | **Mean square** | ***F*** | ***p*(same)** |
| **Between groups** | 51.0245 | 2 | 25.5122 | 10.55 | 0.0001359 |
| **Within groups** | 130.621 | 54 | 2.41891 |  |  |

One-way ANOVA results for the greatest length (GL) of the female carpometacarpus. Taking into account only modern material.

| **Table 10**, Female carpometacarpus GL ANOVA – Tukey’s pairwise. No Swedish female carpometacarpus data was available. | | | | |
| --- | --- | --- | --- | --- |
|  | **Norway** | **Denmark** | **Finland** |  |
| **Norway** | - | 0.481 | 0.100 |  |
| **Denmark** | 1.643 | - | >.001* |  |
| **Finland** | 2.965 | 6.385 | - |  |

# Male carpometacarpus Greatest Length (GL)

Male carpometacarpus GL shows the groups are normally distributed (Shapiro-Wilk p = 0.250). The Levene’s test for homogeneity of variance reports a p value of 0.660, this means that the variances are equal, and the assumptions of a one-way ANOVA are met. Table 11 presents the results of the one-way ANOVA in standard form and Table 12 shows the Tukey’s pairwise results.

| **Table 11**, Male carpometacarpus GL one-way ANOVA | | | | | |
| --- | --- | --- | --- | --- | --- |
|  | **Sum of sqrs** | **df** | **Mean square** | ***F*** | ***p*(same)** |
| **Between groups** | 81.8836 | 2 | 40.9418 | 18.13 | 5.545E-07 |
| **Within groups** | 146.814 | 65 | 2.25867 |  |  |

One-way ANOVA results for the greatest length (GL) of the male carpometacarpus. Taking into account only modern material.

| **Table 12**, Male carpometacarpus GL ANOVA – Tukey’s pairwise. No Swedish male carpometacarpus data was available. | | | | |
| --- | --- | --- | --- | --- |
|  | **Norway** | **Denmark** | **Finland** |  |
| **Norway** | - | >.001* | 0.495 |  |
| **Denmark** | 6.141 | - | >.001* |  |
| **Finland** | 1.607 | 8.02 | - |  |

# Female femur Greatest Length (GL)

Female femur GL shows the groups are normally distributed (Shapiro-Wilk p = 0.083). The Levene’s test for homogeneity of variance reports a p value of 0.04, this means that the variances are unequal, this compromises the assumptions of a one-way ANOVA. As a result, Welch’s F test (in the case of unequal variances) was consulted and included in Table 13 along with the one-way ANOVA results, as the p value of the Welch’s test was significant (p=0.01) it was possible to continue to Tukey’s pairwise (Table 14). Table 13 presents the results of the one-way ANOVA in standard form and Table 14 shows the Tukey’s pairwise results.

| **Table 13**, Female femur GL one-way ANOVA | | | | | |
| --- | --- | --- | --- | --- | --- |
|  | **Sum of sqrs** | **df** | **Mean square** | ***F*** | ***p*(same)** |
| **Between groups** | 68.3521 | 3 | 22.784 | 4.498 | 0.005376 |
| **Within groups** | 481.233 | 95 | 5.06562 |  |  |
| **Welch *F* test** |  | 23.36 |  | 4.396 | 0.01367 |

One-way ANOVA results for the greatest length (GL) of the female femur. Taking into account only modern material.

| **Table 14**, Female femur GL ANOVA – Tukey’s pairwise | | | |  |  |  |
| --- | --- | --- | --- | --- | --- | --- |
|  | **Norway** | **Sweden** | **Denmark** | **Finland** |  |  |
| **Norway** | - | 0.867 | 0.109 | 0.903 |  |  |
| **Sweden** | 1.093 | - | 0.138 | 0.983 |  |  |
| **Denmark** | 3.228 | 3.073 | - | 0.008* |  |  |
| **Finland** | 0.968 | 0.520 | 4.609 | - |  |  |

# Male femur Greatest Length (GL)

Male femur GL shows the groups are normally distributed (Shapiro-Wilk p = 0.852). The Levene’s test for homogeneity of variance reports a p value of 0.06, so there is no significant difference. This means the assumptions of the ANOVA are safely met. Table 15 presents the results of the one-way ANOVA in standard form and Table 16 shows the Tukey’s pairwise results.

| **Table 15**, Male femur GL one-way ANOVA | | | | | |
| --- | --- | --- | --- | --- | --- |
|  | **Sum of sqrs** | **df** | **Mean square** | ***F*** | ***p*(same)** |
| **Between groups** | 87.3141 | 3 | 29.1047 | 6.745 | 0.0002996 |
| **Within groups** | 530.758 | 123 | 4.3151 |  |  |

One-way ANOVA results for the greatest length (GL) of the male femur. Taking into account only modern material.

| **Table 16**, Male femur GL ANOVA – Tukey’s pairwise | | | |  |  |  |
| --- | --- | --- | --- | --- | --- | --- |
|  | **Norway** | **Sweden** | **Denmark** | **Finland** |  |  |
| **Norway** | - | 0.999 | 0.004* | 0.906 |  |  |
| **Sweden** | 0.147 | - | 0.028* | 0.899 |  |  |
| **Denmark** | 4.932 | 3.995 | - | 0.002* |  |  |
| **Finland** | 0.955 | 0.981 | 5.156 | - |  |  |

# Female tibiotarsus Greatest Length (GL)

Female tibiotarsus GL shows the groups are normally distributed (Shapiro-Wilk p = 0.408). The Levene’s test for homogeneity of variance reports a p value of 0.108, this means that the variances are equal, and the assumptions of a one-way ANOVA are met. Table 17 presents the results of the one-way ANOVA in standard form and Table 18 shows the Tukey’s pairwise results.

| **Table 17**, Female tibiotarsus GL one-way ANOVA | | | | | |
| --- | --- | --- | --- | --- | --- |
|  | **Sum of sqrs** | **df** | **Mean square** | ***F*** | ***p*(same)** |
| **Between groups** | 32.0995 | 2 | 16.0497 | 2.658 | 0.07844 |
| **Within groups** | 356.2 | 59 | 6.03729 |  |  |

One-way ANOVA results for the greatest length (GL) of the female tibiotarsus. Taking into account only modern material.

| **Table 18**, Female tibiotarsus GL ANOVA – Tukey’s pairwise. No Swedish female tibiotarsus data was available. | | | | |
| --- | --- | --- | --- | --- |
|  | **Norway** | **Denmark** | **Finland** |  |
| **Norway** | - | 0.986 | 0.230 |  |
| **Denmark** | 0.230 | - | 0.111 |  |
| **Finland** | 2.345 | 2.891 | - |  |

# Male tibiotarsus Greatest Length (GL)

Male tibiotarsus GL shows the groups are normally distributed (Shapiro-Wilk p = 0.812). The Levene’s test for homogeneity of variance reports a p value of 0.891, this means that the variances are equal, and the assumptions of a one-way ANOVA are met. Table 19 presents the results of the one-way ANOVA in standard form and Table 20 shows the Tukey’s pairwise results.

| **Table 19**, Male tibiotarsus GL one-way ANOVA | | | | | |
| --- | --- | --- | --- | --- | --- |
|  | **Sum of sqrs** | **df** | **Mean square** | ***F*** | ***p*(same)** |
| **Between groups** | 89.1457 | 2 | 44.5728 | 8.3 | 0.0006092 |
| **Within groups** | 354.454 | 66 | 5.37051 |  |  |

One-way ANOVA results for the greatest length (GL) of the male tibiotarsus. Taking into account only modern material.

| **Table 20**, Male tibiotarsus GL ANOVA – Tukey’s pairwise. No Swedish male tibiotarsus data was available. | | | | |
| --- | --- | --- | --- | --- |
|  | **Norway** | **Denmark** | **Finland** |  |
| **Norway** | - | 0.009* | 0.731 |  |
| **Denmark** | 4.299 | - | >.001* |  |
| **Finland** | 1.069 | 5.402 | - |  |

# Female tarsometatarsus Greatest Length (GL)

Female tarsometatarsus GL shows the groups are normally distributed (Shapiro-Wilk p = 0.298). The Levene’s test for homogeneity of variance reports a p value of 0.307, this means that the variances are equal, and the assumptions of a one-way ANOVA are met. Table 21 presents the results of the one-way ANOVA in standard form and Table 22 shows the Tukey’s pairwise results.

| **Table 21**, Female tarsometatarsus GL one-way ANOVA | | | | | |
| --- | --- | --- | --- | --- | --- |
|  | **Sum of sqrs** | **df** | **Mean square** | ***F*** | ***p*(same)** |
| **Between groups** | 47.7638 | 2 | 23.8819 | 6.721 | 0.00237 |
| **Within groups** | 206.094 | 58 | 3.55335 |  |  |

One-way ANOVA results for the greatest length (GL) of the female tarsometatarsus. Taking into account only modern material.

| **Table 22**, Female tarsometatarsus GL ANOVA – Tukey’s pairwise. No Swedish female tarsometatarsus data was available. | | | | |
| --- | --- | --- | --- | --- |
|  | **Norway** | **Denmark** | **Finland** |  |
| **Norway** | - | 0.976 | 0.023* |  |
| **Denmark** | 0.295 | - | 0.006* |  |
| **Finland** | 3.841 | 4.531 | - |  |

# Male tarsometatarsus Greatest Length (GL)

Male tarsometatarsus GL shows the groups are normally distributed (Shapiro-Wilk p = 0.798). The Levene’s test for homogeneity of variance reports a p value of 0.571, this means that the variances are equal, and the assumptions of a one-way ANOVA are met. Table 23 presents the results of the one-way ANOVA in standard form and Table 24 shows the Tukey’s pairwise results.

| **Table 23**, Male tarsometatarsus GL one-way ANOVA | | | | | |
| --- | --- | --- | --- | --- | --- |
|  | **Sum of sqrs** | **df** | **Mean square** | ***F*** | ***p*(same)** |
| **Between groups** | 65.6032 | 2 | 32.8016 | 7.91 | 0.0008517 |
| **Within groups** | 265.408 | 64 | 4.14701 |  |  |

One-way ANOVA results for the greatest length (GL) of the male tarsometatarsus. Taking into account only modern material.

| **Table 24**, Male tarsometatarsus GL ANOVA – Tukey’s pairwise. No Swedish male tarsometatarsus data was available. | | | | |
| --- | --- | --- | --- | --- |
|  | **Norway** | **Denmark** | **Finland** |  |
| **Norway** | - | 0.042* | 0.407 |  |
| **Denmark** | 3.498 | - | >.001* |  |
| **Finland** | 1.822 | 5.515 | - |  |
