# Supplementary material for: A long‐term study of size variation in Northern Goshawk Accipiter gentilis across Scandinavia, with a focus on Norway
Source: Ecol Evol. 2023 Dec 7;13(12):e10789. doi: 10.1002/ece3.10789 (PMC10701624; doi:10.1002/ece3.10789)
Supplement: Supplementary file 2 — File S2. [file ECE3-13-e10789-s002.docx]

**Supporting Information File 2 (SIF2)**. PCA results.

**Table 1.** Principal Components Analysis (PCAs) results for modern male and female *Accipiter gentilis gentilis*. All modern material was included here (Norway, Denmark, Sweden and Finland). Archaeological material was not included in this PCA. Specimens were not included in this PCA if more than one measurement was missing, so as not to skew any results.

| **Component** | **Eigenvalue** | **% Variance** | **Loadings** | **PC1** | **PC2** | **PC3** | **PC4** | **PC5** | **PC6** |
| --- | --- | --- | --- | --- | --- | --- | --- | --- | --- |
| *Analysis 1, Humerus PCA results, n = 188 (113 males and 75 females)* | | | | | | | | | |
| **PC1** | 51.162 | 98.98 | GL | 0.944 | -0.327 | -0.016 | 0.037 | -0.015 | - |
| **PC2** | 0.409 | 0.78 | Bp | 0.239 | 0.751 | -0.536 | 0.303 | -0.018 | - |
| **PC3** | 0.056 | 0.11 | SC | 0.081 | 0.271 | 0.743 | 0.568 | -0.212 | - |
| **PC4** | 0.048 | 0.09 | Bd | 0.202 | 0.486 | 0.344 | -0.764 | -0.144 | - |
| **PC5** | 0.022 | 0.04 | KB | 0.066 | 0.141 | 0.204 | 0.017 | 0.966 | - |
| *Analysis 2, Ulna PCA results, n = 128 (69 males and 59 females)* | | | | | | | | | |
| **PC1** | 54.485 | 98.88 | GL | 0.971 | -0.235 | -0.030 | 0.003 | -0.012 | -0.011 |
| **PC2** | 0.440 | 0.80 | Dip | 0.123 | 0.613 | -0.767 | 0.101 | 0.019 | -0.099 |
| **PC3** | 0.089 | 0.16 | Bp | 0.131 | 0.472 | 0.438 | -0.228 | 0.577 | -0.447 |
| **PC4** | 0.038 | 0.07 | Tp | 0.096 | 0.327 | 0.139 | -0.206 | 0.181 | 0.889 |
| **PC5** | 0.036 | 0.06 | SC | 0.055 | 0.200 | 0.281 | 0.932 | 0.040 | 0.084 |
| **PC6** | 0.016 | 0.03 | Did | 0.108 | 0.446 | 0.347 | -0.163 | -0.795 | -0.106 |
| *Analysis 3, Carpometacarpus PCA results, n = 126 (69 males and 57 females)* | | | | | | | | |  |
| **PC1** | 19.951 | 98.04 | GL | 0.942 | -0.294 | -0.118 | 0.111 | - | - |
| **PC2** | 0.187 | 0.92 | Bp | 0.264 | 0.486 | 0.128 | -0.823 | - | - |
| **PC3** | 0.125 | 0.61 | Did | 0.169 | 0.819 | -0.217 | 0.504 | - | - |
| **PC4** | 0.087 | 0.43 | HS | 0.118 | 0.085 | 0.961 | 0.237 | - | - |
| *Analysis 4, Femur PCA results, n = 226 (127 males and 99 females)* | | | | | | | | | |
| **PC1** | 43.838 | 98.13 | GL | 0.895 | -0.440 | -0.039 | -0.056 | 0.022 | 0.003 |
| **PC2** | 0.464 | 1.04 | Bp | 0.241 | 0.611 | -0.519 | -0.538 | 0.096 | -0.015 |
| **PC3** | 0.159 | 0.36 | Dp | 0.141 | 0.259 | -0.017 | 0.516 | 0.757 | -0.271 |
| **PC4** | 0.092 | 0.21 | SC | 0.101 | 0.175 | -0.191 | 0.397 | -0.041 | 0.873 |
| **PC5** | 0.064 | 0.14 | Bd | 0.275 | 0.460 | 0.103 | 0.444 | -0.628 | -0.333 |
| **PC6** | 0.057 | 0.13 | Dd | 0.186 | 0.352 | 0.826 | -0.293 | 0.146 | 0.229 |
| *Analysis 5, Tibiotarsus PCA results, n = 133 (71 males and 62 females)* | | | | | | | | | |
| **PC1** | 63.797 | 98.77 | GL | 0.937 | -0.346 | 0.010 | -0.028 | 0.036 | 0.001 |
| **PC2** | 0.458 | 0.71 | Dip | 0.226 | 0.623 | -0.321 | -0.488 | -0.179 | -0.433 |
| **PC3** | 0.187 | 0.29 | Bp | 0.158 | 0.438 | 0.879 | 0.058 | -0.086 | 0.029 |
| **PC4** | 0.066 | 0.10 | SC | 0.075 | 0.303 | -0.081 | -0.156 | 0.864 | 0.353 |
| **PC5** | 0.050 | 0.08 | Bd | 0.167 | 0.378 | -0.265 | 0.856 | 0.047 | -0.157 |
| **PC6** | 0.035 | 0.05 | Dd | 0.113 | 0.258 | -0.219 | -0.029 | -0.458 | 0.814 |
| *Analysis 6, Tarsometatarsus PCA results, n = 128 (68 males and 60 females)* | | | | | | | | | |
| **PC1** | 30.608 | 98.13 | GL | 0.922 | -0.386 | 0.009 | -0.002 | - | - |
| **PC2** | 0.380 | 1.22 | Bp | 0.247 | 0.607 | 0.649 | -0.386 | - | - |
| **PC3** | 0.114 | 0.37 | SC | 0.127 | 0.302 | 0.216 | 0.920 | - | - |
| **PC4** | 0.090 | 0.29 | Bd | 0.268 | 0.625 | -0.730 | -0.071 | - | - |
