# Supplementary material for: A long‐term study of size variation in Northern Goshawk Accipiter gentilis across Scandinavia, with a focus on Norway
Source: Ecol Evol. 2023 Dec 7;13(12):e10789. doi: 10.1002/ece3.10789 (PMC10701624; doi:10.1002/ece3.10789)
Supplement: Supplementary file 3 — File S3. [file ECE3-13-e10789-s005.docx]

**Supporting Information File 3 (SIF3)**. Size range tables for modern Scandinavian *A. g. gentilis* skeletal elements. Medieval specimens from Norway have also been included for the humerus, ulna, carpometacarpus, femur, tibiotarsus and tarsometatarsus. Diagrams and detailed descriptions of how these measurements were taken can be found in Von den Driesch (1976), unless noted in the caption.

**Table 1.** Modern size ranges for *A. g. gentilis* cranial measurements. The *A. g. gentilis* data is made up of modern specimens from Norway, Sweden and Denmark. The *A. g. gentilis* (Finland) data represents only modern specimens from Finland (not including Lapland). Abbreviations: GL = greatest length, GB = greatest breadth, SBO = smallest breadth between the orbits, LI = length of the Incisivum. LP = length from the Protuberantia occipitalis externa to the most aboral point of the processus frontales, GH = greatest height, CBL = condylobasal length. The measurements are in mm.

|  | **Sex** | **No. specimens** | **Observed range (mm)** | **Mean ± standard error** |
| --- | --- | --- | --- | --- |
| **Cranium GL** |  |  |  |  |
| *A. g. gentilis* (Norway & Sweden) | ♂ | 20 | 69.91 – 76.20 | 72.91 ± 0.33 |
| *A. g. gentilis* (Norway & Sweden) | ♀ | 8 | 76.39 – 80.87 | 78.07 ± 0.50 |
| *A. g. gentilis* (Denmark) | ♂ | 18 | 64.22 – 75.51 | 71.40 ± 0.63 |
| *A. g. gentilis* (Denmark) | ♀ | 19 | 73.39 – 83.85 | 77.88 ± 0.73 |
| *A. g. gentilis* (Finland) | ♂ | 13 | 70.91 – 76.57 | 74.15 ± 0.41 |
| *A. g. gentilis* (Finland) | ♀ | 21 | 76.46 – 83.19 | 80.38 ± 0.34 |
| **Cranium GB** |  |  |  |  |
| *A. g. gentilis* (Norway & Sweden) | ♂ | 21 | 40.67 – 43.56 | 42.41 ± 0.14 |
| *A. g. gentilis* (Norway & Sweden) | ♀ | 7 | 43.69 – 46.04 | 44.82 ± 0.35 |
| *A. g. gentilis* (Denmark) | ♂ | 18 | 38.69 – 45.18 | 41.33 ± 0.40 |
| *A. g. gentilis* (Denmark) | ♀ | 15 | 41.99 – 48.02 | 44.02 ± 0.48 |
| *A. g. gentilis* (Finland) | ♂ | 13 | 39.46 – 44.40 | 42.57 ± 0.33 |
| *A. g. gentilis* (Finland) | ♀ | 20 | 42.96 – 46.99 | 45.14 ± 0.26 |
| **Cranium SBO** |  |  |  |  |
| *A. g. gentilis* (Norway & Sweden) | ♂ | 22 | 9.98 – 12.10 | 10.84 ± 0.12 |
| *A. g. gentilis* (Norway & Sweden) | ♀ | 8 | 10.87 – 13.43 | 12.15 ± 0.31 |
| *A. g. gentilis* (Denmark) | ♂ | 25 | 9.94 – 14.49 | 10.93 ± 0.21 |
| *A. g. gentilis* (Denmark) | ♀ | 20 | 10.40 – 15.37 | 12.34 ± 0.24 |
| *A. g. gentilis* (Finland) | ♂ | 13 | 9.33 – 11.73 | 10.71 ± 0.21 |
| *A. g. gentilis* (Finland) | ♀ | 21 | 10.73 – 13.18 | 12.06 ± 0.14 |
| **Cranium LI** |  |  |  |  |
| *A. g. gentilis* (Norway & Sweden) | ♂ | 21 | 25.12 – 30.37 | 28.27 ± 0.28 |
| *A. g. gentilis* (Norway & Sweden) | ♀ | 8 | 29.45 – 33.86 | 32.15 ± 0.53 |
| *A. g. gentilis* (Denmark) | ♂ | 21 | 25.05 – 30.57 | 27.85 ± 0.38 |
| *A. g. gentilis* (Denmark) | ♀ | 19 | 30.07 – 37.86 | 32.53 ± 0.49 |
| *A. g. gentilis* (Finland) | ♂ | 13 | 27.20 – 35.08 | 31.53 ± 0.62 |
| *A. g. gentilis* (Finland) | ♀ | 21 | 30.34 – 40.74 | 35.51 ± 0.54 |
| **Cranium LP** |  |  |  |  |
| *A. g. gentilis* (Norway & Sweden) | ♂ | 21 | 49.35 – 54.53 | 51.43 ± 0.29 |
| *A. g. gentilis* (Norway & Sweden) | ♀ | 8 | 50.36 – 55.22 | 53.52 ± 0.53 |
| *A. g. gentilis* (Denmark) | ♂ | 20 | 47.77 – 52.63 | 50.69 ± 0.30 |
| *A. g. gentilis* (Denmark) | ♀ | 20 | 51.09 – 56.89 | 53.93 ± 0.35 |
| *A. g. gentilis* (Finland) | ♂ | 13 | 50.71 – 53.00 | 52.07 ± 0.18 |
| *A. g. gentilis* (Finland) | ♀ | 21 | 52.78 – 55.97 | 54.36 ± 0.23 |
| **Cranium GH** |  |  |  |  |
| *A. g. gentilis* (Norway & Sweden) | ♂ | 21 | 30.13 – 33.70 | 32.35 ± 0.18 |
| *A. g. gentilis* (Norway & Sweden) | ♀ | 8 | 32.87 – 34.23 | 33.56 ± 0.15 |
| *A. g. gentilis* (Denmark) | ♂ | 20 | 28.91 – 32.86 | 31.20 ± 0.25 |
| *A. g. gentilis* (Denmark) | ♀ | 19 | 28.75 – 35.28 | 32.31 ± 0.34 |
| *A. g. gentilis* (Finland) | ♂ | 13 | 32.00 – 33.35 | 32.90 ± 0.12 |
| *A. g. gentilis* (Finland) | ♀ | 20 | 32.26 – 34.87 | 34.00 ± 0.14 |
| **Cranium CBL** |  |  |  |  |
| *A. g. gentilis* (Norway & Sweden) | ♂ | 20 | 57.97 – 65.43 | 61.13 ± 0.42 |
| *A. g. gentilis* (Norway & Sweden) | ♀ | 8 | 64.25 – 68.89 | 66.37 ± 0.54 |
| *A. g. gentilis* (Denmark) | ♂ | 17 | 52.01 – 64.26 | 59.69 ± 0.70 |
| *A. g. gentilis* (Denmark) | ♀ | 19 | 58.59 – 72.35 | 65.83 ± 0.81 |
| *A. g. gentilis* (Finland) | ♂ | 13 | 58.29 – 63.44 | 61.53 ± 0.37 |
| *A. g. gentilis* (Finland) | ♀ | 21 | 65.25 – 70.96 | 68.39 ± 0.30 |

**Table 2.** Modern size ranges for *A. g. gentilis* mandible measurements. The *A. g. gentilis* data is made up of modern specimens from Norway, Sweden and Denmark. The *A. g. gentilis* (Finland) data represents only modern specimens from Finland (not including Lapland). Abbreviations: GL = greatest length, LaF = length from the most aboral point of the articular surface, LS = length of symphysis. The measurements are in mm.

|  | **Sex** | **No. specimens** | **Observed range (mm)** | **Mean ± standard error** |
| --- | --- | --- | --- | --- |
| **Mandible GL** |  |  |  |  |
| *A. g. gentilis* (Norway & Sweden) | ♂ | 20 | 46.31 – 50.62 | 48.46 ± 0.24 |
| *A. g. gentilis* (Norway & Sweden) | ♀ | 8 | 52.12 – 55.65 | 53.92 ± 0.44 |
| *A. g. gentilis* (Denmark) | ♂ | 23 | 42.45 – 53.79 | 46.35 ± 0.45 |
| *A. g. gentilis* (Denmark) | ♀ | 19 | 49.77 – 57.36 | 52.22 ± 0.49 |
| *A. g. gentilis* (Finland) | ♂ | 12 | 47.77 – 52.10 | 49.94 ± 0.36 |
| *A. g. gentilis* (Finland) | ♀ | 21 | 51.22 – 59.10 | 55.37 ± 0.44 |
| **Mandible LaF** |  |  |  |  |
| *A. g. gentilis* (Norway & Sweden) | ♂ | 20 | 42.42 – 46.65 | 44.61 ± 0.26 |
| *A. g. gentilis* (Norway & Sweden) | ♀ | 8 | 47.43 – 51.82 | 49.61 ± 0.56 |
| *A. g. gentilis* (Denmark) | ♂ | 23 | 40.00 – 49.07 | 42.65 ± 0.37 |
| *A. g. gentilis* (Denmark) | ♀ | 19 | 45.26 – 50.73 | 47.49 ± 0.42 |
| *A. g. gentilis* (Finland) | ♂ | 12 | 43.13 – 46.67 | 45.10 ± 0.31 |
| *A. g. gentilis* (Finland) | ♀ | 21 | 47.06 – 50.17 | 50.17 ± 0.39 |
| **Mandible LS** |  |  |  |  |
| *A. g. gentilis* (Norway & Sweden) | ♂ | 20 | 8.64 – 9.75 | 9.17 ± 0.07 |
| *A. g. gentilis* (Norway & Sweden) | ♀ | 8 | 9.95 – 10.94 | 10.33 ± 0.12 |
| *A. g. gentilis* (Denmark) | ♂ | 24 | 8.17 – 9.53 | 8.92 ± 0.08 |
| *A. g. gentilis* (Denmark) | ♀ | 19 | 9.51 – 10.93 | 10.11 ± 0.08 |
| *A. g. gentilis* (Finland) | ♂ | 12 | 8.64 – 12.42 | 10.92 ± 0.35 |
| *A. g. gentilis* (Finland) | ♀ | 21 | 9.51 – 13.61 | 11.54 ± 0.30 |

**Table 3.** Modern size ranges for *A. g. gentilis* sternum measurements. The *A. g. gentilis* data is made up of modern specimens from Norway, Sweden and Denmark. The *A. g. gentilis* (Finland) data represents only modern specimens from Finland (not including Lapland). Abbreviations: Lm = length from the Manubrium sterni to the Metasternum, dL = dorsal length, LC = length of the Crista sterni, SBF = smallest breadth between the facets. The measurements are in mm.

|  | **Sex** | **No. specimens** | **Observed range (mm)** | **Mean ± standard error** |
| --- | --- | --- | --- | --- |
| **Sternum Lm** |  |  |  |  |
| *A. g. gentilis* (Norway & Sweden) | ♂ | 43 | 75.21 – 86.84 | 79.64 ± 0.35 |
| *A. g. gentilis* (Norway & Sweden) | ♀ | 26 | 82.72 – 94.08 | 88.39 ± 0.61 |
| *A. g. gentilis* (Denmark) | ♂ | 42 | 71.26 – 89.21 | 78.09 ± 0.70 |
| *A. g. gentilis* (Denmark) | ♀ | 31 | 79.20 – 94.22 | 86.06 ± 0.82 |
| *A. g. gentilis* (Finland) | ♂ | 12 | 76.96 – 85.72 | 82.10 ± 0.72 |
| *A. g. gentilis* (Finland) | ♀ | 20 | 88.14 – 94.82 | 91.08 ± 0.43 |
| **Sternum dL** |  |  |  |  |
| *A. g. gentilis* (Norway & Sweden) | ♂ | 43 | 67.29 – 78.43 | 71.71 ± 0.32 |
| *A. g. gentilis* (Norway & Sweden) | ♀ | 26 | 75.23 – 84.95 | 80.03 ± 0.56 |
| *A. g. gentilis* (Denmark) | ♂ | 42 | 63.83 – 80.93 | 70.32 ± 0.65 |
| *A. g. gentilis* (Denmark) | ♀ | 31 | 71.64 – 84.94 | 78.03 ± 0.73 |
| *A. g. gentilis* (Finland) | ♂ | 12 | 68.68 – 77.49 | 73.84 ± 0.68 |
| *A. g. gentilis* (Finland) | ♀ | 20 | 79.32 – 86.37 | 82.47 ± 0.42 |
| **Sternum LC** |  |  |  |  |
| *A. g. gentilis* (Norway & Sweden) | ♂ | 43 | 77.29 – 88.02 | 80.55 ± 0.34 |
| *A. g. gentilis* (Norway & Sweden) | ♀ | 26 | 81.20 – 93.58 | 88.09 ± 0.65 |
| *A. g. gentilis* (Denmark) | ♂ | 42 | 69.66 – 91.17 | 78.77 ± 0.74 |
| *A. g. gentilis* (Denmark) | ♀ | 31 | 79.55 – 94.42 | 86.45 ± 0.80 |
| *A. g. gentilis* (Finland) | ♂ | 12 | 78.54 – 85.89 | 83.27 ± 0.63 |
| *A. g. gentilis* (Finland) | ♀ | 20 | 86.65 – 95.36 | 90.65 ± 0.51 |
| **Sternum SBF** |  |  |  |  |
| *A. g. gentilis* (Norway & Sweden) | ♂ | 43 | 31.14 – 38.62 | 34.26 ± 0.26 |
| *A. g. gentilis* (Norway & Sweden) | ♀ | 26 | 34.05 – 41.56 | 38.13 ± 0.37 |
| *A. g. gentilis* (Denmark) | ♂ | 42 | 29.34 – 37.65 | 33.32 ± 0.26 |
| *A. g. gentilis* (Denmark) | ♀ | 31 | 34.20 – 42.28 | 37.45 ± 0.35 |
| *A. g. gentilis* (Finland) | ♂ | 12 | 33.24 – 37.44 | 35.44 ± 0.38 |
| *A. g. gentilis* (Finland) | ♀ | 20 | 36.10 – 41.29 | 38.96 ± 0.31 |

**Table 4.** Modern size ranges for *A. g. gentilis* coracoid measurements. The *A. g. gentilis* data is made up of modern specimens from Norway, Sweden and Denmark. The *A. g. gentilis* (Finland) data represents only modern specimens from Finland (not including Lapland). Abbreviations: GL = greatest length, Lm = medial length, Bb = basal breadth, BF = breadth of the Facies articularis basalis. The measurements are in mm.

|  | **Sex** | **No. specimens** | **Observed range (mm)** | **Mean ± standard error** |
| --- | --- | --- | --- | --- |
| **Coracoid GL** |  |  |  |  |
| *A. g. gentilis* (Norway & Sweden) | ♂ | 61 | 44.19 – 50.84 | 47.57 ± 0.18 |
| *A. g. gentilis* (Norway & Sweden) | ♀ | 29 | 51.60 – 56.56 | 53.54 ± 0.23 |
| *A. g. gentilis* (Denmark) | ♂ | 50 | 42.17 – 51.03 | 45.88 ± 0.29 |
| *A. g. gentilis* (Denmark) | ♀ | 38 | 48.24 – 58.98 | 52.33 ± 0.41 |
| *A. g. gentilis* (Finland) | ♂ | 21 | 45.88 – 51.00 | 48.06 ± 0.30 |
| *A. g. gentilis* (Finland) | ♀ | 30 | 50.66 – 56.78 | 53.55 ± 0.25 |
| **Coracoid Lm** |  |  |  |  |
| *A. g. gentilis* (Norway & Sweden) | ♂ | 61 | 41.88 – 48.18 | 44.60 ± 0.18 |
| *A. g. gentilis* (Norway & Sweden) | ♀ | 29 | 47.97 – 53.05 | 50.13 ± 0.25 |
| *A. g. gentilis* (Denmark) | ♂ | 50 | 40.41 – 48.04 | 43.28 ± 0.25 |
| *A. g. gentilis* (Denmark) | ♀ | 38 | 45.48 – 55.02 | 48.91 ± 0.36 |
| *A. g. gentilis* (Finland) | ♂ | 21 | 42.83 – 47.94 | 45.18 ± 0.28 |
| *A. g. gentilis* (Finland) | ♀ | 30 | 47.83 – 53.47 | 50.49 ± 0.22 |
| **Coracoid Bb** |  |  |  |  |
| *A. g. gentilis* (Norway & Sweden) | ♂ | 61 | 18.56 – 22.68 | 20.26 ± 0.10 |
| *A. g. gentilis* (Norway & Sweden) | ♀ | 29 | 19.84 – 24.47 | 22.91 ± 0.18 |
| *A. g. gentilis* (Denmark) | ♂ | 52 | 16.27 – 23.43 | 19.73 ± 0.18 |
| *A. g. gentilis* (Denmark) | ♀ | 38 | 19.51 – 26.34 | 22.40 ± 0.24 |
| *A. g. gentilis* (Finland) | ♂ | 21 | 19.09 – 22.82 | 20.92 ± 0.22 |
| *A. g. gentilis* (Finland) | ♀ | 30 | 21.48 – 25.33 | 23.49 ± 0.19 |
| **Coracoid BF** |  |  |  |  |
| *A. g. gentilis* (Norway & Sweden) | ♂ | 61 | 13.76 – 17.19 | 15.20 ± 0.09 |
| *A. g. gentilis* (Norway & Sweden) | ♀ | 29 | 15.96 – 19.03 | 17.19 ± 0.14 |
| *A. g. gentilis* (Denmark) | ♂ | 52 | 11.88 – 18.61 | 14.64 ± 0.15 |
| *A. g. gentilis* (Denmark) | ♀ | 38 | 14.49 – 20.11 | 16.59 ± 0.19 |
| *A. g. gentilis* (Finland) | ♂ | 21 | 14.28 – 17.18 | 15.75 ± 0.15 |
| *A. g. gentilis* (Finland) | ♀ | 30 | 15.78 – 19.61 | 17.73 ± 0.17 |

**Table 5.** Modern size ranges for *A. g. gentilis* scapula measurements. The *A. g. gentilis* data is made up of modern specimens from Norway, Sweden and Denmark. The *A. g. gentilis* (Finland) data represents only modern specimens from Finland (not including Lapland). Abbreviations: GL = greatest length, Dic = cranial diagonal, KC = smallest breadth of the Collum scapulae (measurement taken from Otto, 1981). The measurements are in mm.

|  | **Sex** | **No. specimens** | **Observed range (mm)** | **Mean ± standard error** |
| --- | --- | --- | --- | --- |
| **Scapula GL** |  |  |  |  |
| *A. g. gentilis* (Norway & Sweden) | ♂ | 42 | 59.42 – 65.50 | 62.81 ± 0.24 |
| *A. g. gentilis* (Norway & Sweden) | ♀ | 26 | 69.24 – 74.37 | 71.88 ± 0.34 |
| *A. g. gentilis* (Denmark) | ♂ | 39 | 54.42 – 66.75 | 60.81 ± 0.51 |
| *A. g. gentilis* (Denmark) | ♀ | 30 | 65.64 – 75.36 | 69.06 ± 0.51 |
| *A. g. gentilis* (Finland) | ♂ | 20 | 60.16 – 65.96 | 63.50 ± 0.35 |
| *A. g. gentilis* (Finland) | ♀ | 27 | 69.81 – 76.50 | 72.52 ± 0.34 |
| **Scapula Dic** |  |  |  |  |
| *A. g. gentilis* (Norway & Sweden) | ♂ | 42 | 13.31 – 15.19 | 14.20 ± 0.07 |
| *A. g. gentilis* (Norway & Sweden) | ♀ | 26 | 14.74 – 18.24 | 16.51 ± 0.13 |
| *A. g. gentilis* (Denmark) | ♂ | 42 | 12.25 – 15.34 | 13.82 ± 0.12 |
| *A. g. gentilis* (Denmark) | ♀ | 31 | 14.71 – 17.72 | 16.00 ± 0.14 |
| *A. g. gentilis* (Finland) | ♂ | 21 | 13.68 – 15.23 | 14.35 ± 0.09 |
| *A. g. gentilis* (Finland) | ♀ | 30 | 15.63 – 17.91 | 16.84 ± 0.10 |
| **Scapula KC** |  |  |  |  |
| *A. g. gentilis* (Norway & Sweden) | ♂ | 42 | 4.66 – 5.53 | 5.05 ± 0.03 |
| *A. g. gentilis* (Norway & Sweden) | ♀ | 26 | 5.36 – 6.61 | 5.79 ± 0.06 |
| *A. g. gentilis* (Denmark) | ♂ | 42 | 4.41 – 5.89 | 5.06 ± 0.04 |
| *A. g. gentilis* (Denmark) | ♀ | 31 | 5.28 – 6.22 | 5.70 ± 0.05 |
| *A. g. gentilis* (Finland) | ♂ | 21 | 4.79 – 5.73 | 5.22 ± 0.05 |
| *A. g. gentilis* (Finland) | ♀ | 30 | 5.62 – 6.86 | 6.01 ± 0.05 |

**Table 6.** Modern and archaeological size ranges for *A. g. gentilis* humerus measurements. The *A. g. gentilis* data is made up of modern specimens from Norway, Sweden and Denmark. The *A. g. gentilis* (Finland) data represents only modern specimens from Finland (not including Lapland). The *A. gentilis* (Medieval) data is from Northern Goshawk recovered from Medieval dated contexts in Norway. Abbreviations: GL = greatest length, Bp = breadth of the proximal end, SC = smallest breadth of the corpus, Bd = breadth of the distal end, KB = smallest depth of the distal shaft (measurement taken from Kraft, 1972). The measurements are in mm.

|  | **Sex** | **No. specimens** | **Observed range (mm)** | **Mean ± standard error** |
| --- | --- | --- | --- | --- |
| **Humerus GL** |  |  |  |  |
| *A. g. gentilis* (Norway & Sweden) | ♂ | 44 | 88.49 – 98.67 | 93.28 ± 0.30 |
| *A. g. gentilis* (Norway & Sweden) | ♀ | 15 | 101.92 – 110.83 | 105.92 ± 0.55 |
| *A. g. gentilis* (Denmark) | ♂ | 46 | 86.73 – 97.28 | 91.03 ± 0.38 |
| *A. g. gentilis* (Denmark) | ♀ | 29 | 99.13 – 110.98 | 104.01 ± 0.59 |
| *A. g. gentilis* (Finland) | ♂ | 22 | 89.69 – 97.84 | 93.95 ± 0.48 |
| *A. g. gentilis* (Finland) | ♀ | 30 | 103.11 – 111.41 | 106.44 ± 0.33 |
| *A. gentilis* (Medieval) | ♂ | 2 | 90.00 – 96.46 | 93.23 ± 3.23 |
| *A. gentilis* (Medieval) | ♀ | 5 | 106.10 – 111.92 | 108.31 ± 1.05 |
| **Humerus Bp** |  |  |  |  |
| *A. g. gentilis* (Norway & Sweden) | ♂ | 48 | 20.00 – 22.66 | 21.01 ± 0.09 |
| *A. g. gentilis* (Norway & Sweden) | ♀ | 20 | 23.16 – 25.44 | 24.22 ± 0.15 |
| *A. g. gentilis* (Denmark) | ♂ | 47 | 18.83 – 22.13 | 20.42 ± 0.12 |
| *A. g. gentilis* (Denmark) | ♀ | 33 | 22.17 – 26.06 | 23.73 ± 0.19 |
| *A. g. gentilis* (Finland) | ♂ | 22 | 20.19 – 22.63 | 21.40 ± 0.13 |
| *A. g. gentilis* (Finland) | ♀ | 30 | 23.28 – 25.95 | 24.51 ± 0.13 |
| *A. gentilis* (Medieval) | ♂ | 0 | - | - |
| *A. gentilis* (Medieval) | ♀ | 5 | 23.81 – 24.51 | 24.09 ± 0.14 |
| **Humerus SC** |  |  |  |  |
| *A. g. gentilis* (Norway & Sweden) | ♂ | 44 | 7.12 – 8.08 | 7.61 ± 0.03 |
| *A. g. gentilis* (Norway & Sweden) | ♀ | 15 | 7.79 – 9.06 | 8.56 ± 0.10 |
| *A. g. gentilis* (Denmark) | ♂ | 47 | 6.59 – 8.15 | 7.33 ± 0.05 |
| *A. g. gentilis* (Denmark) | ♀ | 30 | 7.75 – 9.43 | 8.42 ± 0.08 |
| *A. g. gentilis* (Finland) | ♂ | 22 | 7.24 – 8.12 | 7.61 ± 0.05 |
| *A. g. gentilis* (Finland) | ♀ | 30 | 8.07 – 9.52 | 8.72 ± 0.08 |
| *A. gentilis* (Medieval) | ♂ | 4 | 7.10 – 7.76 | 7.36 ± 0.15 |
| *A. gentilis* (Medieval) | ♀ | 13 | 8.25 – 9.16 | 8.83 ± 0.08 |
| **Humerus Bd** |  |  |  |  |
| *A. g. gentilis* (Norway & Sweden) | ♂ | 44 | 16.77 – 19.07 | 17.60 ± 0.06 |
| *A. g. gentilis* (Norway & Sweden) | ♀ | 15 | 19.17 – 21.14 | 20.09 ± 0.16 |
| *A. g. gentilis* (Denmark) | ♂ | 47 | 16.08 – 18.62 | 17.00 ± 0.08 |
| *A. g. gentilis* (Denmark) | ♀ | 30 | 18.50 – 21.24 | 19.76 ± 0.15 |
| *A. g. gentilis* (Finland) | ♂ | 22 | 16.91 – 18.54 | 17.70 ± 0.09 |
| *A. g. gentilis* (Finland) | ♀ | 30 | 19.32 – 21.48 | 20.53 ± 0.10 |
| *A. gentilis* (Medieval) | ♂ | 3 | 16.92 – 17.56 | 17.24 ± 0.18 |
| *A. gentilis* (Medieval) | ♀ | 13 | 19.78 – 20.89 | 20.41 ± 0.11 |
| **Humerus KB** |  |  |  |  |
| *A. g. gentilis* (Norway & Sweden) | ♂ | 44 | 6.06 – 6.94 | 6.37 ± 0.03 |
| *A. g. gentilis* (Norway & Sweden) | ♀ | 15 | 6.83 – 7.66 | 7.26 ± 0.06 |
| *A. g. gentilis* (Denmark) | ♂ | 47 | 5.61 – 6.80 | 6.17 ± 0.04 |
| *A. g. gentilis* (Denmark) | ♀ | 30 | 6.56 – 7.71 | 7.05 ± 0.05 |
| *A. g. gentilis* (Finland) | ♂ | 22 | 6.11 – 6.96 | 6.42 ± 0.04 |
| *A. g. gentilis* (Finland) | ♀ | 30 | 6.99 – 7.76 | 7.32 ± 0.03 |
| *A. gentilis* (Medieval) | ♂ | 3 | 5.89 – 6.53 | 6.16 ± 0.19 |
| *A. gentilis* (Medieval) | ♀ | 10 | 7.19 – 7.76 | 7.42 ± 0.06 |

**Table 7.** Modern size ranges for *A. g. gentilis* radius measurements. The *A. g. gentilis* data is made up of modern specimens from Norway, Sweden and Denmark. The *A. g. gentilis* (Finland) data represents only modern specimens from Finland (not including Lapland). Abbreviations: GL = greatest length, SC = smallest breadth of the corpus, Bd = breadth of the distal end. The measurements are in mm.

|  | **Sex** | **No. specimens** | **Observed range (mm)** | **Mean ± standard error** |
| --- | --- | --- | --- | --- |
| **Radius GL** |  |  |  |  |
| *A. g. gentilis* (Norway & Sweden) | ♂ | 21 | 91.33 – 101.34 | 96.22 ± 0.50 |
| *A. g. gentilis* (Norway & Sweden) | ♀ | 7 | 104.29 – 111.93 | 107.16 ± 1.07 |
| *A. g. gentilis* (Denmark) | ♂ | 25 | 88.96 – 98.93 | 93.65 ± 0.51 |
| *A. g. gentilis* (Denmark) | ♀ | 21 | 102.21 – 112.09 | 106.76 ± 0.65 |
| *A. g. gentilis* (Finland) | ♂ | 22 | 91.83 – 101.31 | 96.98 ± 0.48 |
| *A. g. gentilis* (Finland) | ♀ | 30 | 105.05 – 112.12 | 108.89 ± 0.35 |
| **Radius SC** |  |  |  |  |
| *A. g. gentilis* (Norway & Sweden) | ♂ | 21 | 2.41 – 3.02 | 2.67 ± 0.03 |
| *A. g. gentilis* (Norway & Sweden) | ♀ | 8 | 2.91 – 3.28 | 3.04 ± 0.05 |
| *A. g. gentilis* (Denmark) | ♂ | 27 | 6.66 – 7.91 | 7.34 ± 0.06 |
| *A. g. gentilis* (Denmark) | ♀ | 21 | 2.68 – 3.50 | 3.10 ± 0.04 |
| *A. g. gentilis* (Finland) | ♂ | 22 | 2.47 – 2.90 | 2.68 ± 0.02 |
| *A. g. gentilis* (Finland) | ♀ | 30 | 2.81 – 3.30 | 3.10 ± 0.02 |
| **Radius Bd** |  |  |  |  |
| *A. g. gentilis* (Norway & Sweden) | ♂ | 21 | 7.20 – 7.74 | 7.52 ± 0.03 |
| *A. g. gentilis* (Norway & Sweden) | ♀ | 8 | 8.14 – 8.82 | 8.48 ± 0.09 |
| *A. g. gentilis* (Denmark) | ♂ | 27 | 2.43 – 2.80 | 2.61 ± 0.02 |
| *A. g. gentilis* (Denmark) | ♀ | 21 | 7.66 – 9.60 | 8.54 ± 0.09 |
| *A. g. gentilis* (Finland) | ♂ | 22 | 7.28 – 8.18 | 7.69 ± 0.05 |
| *A. g. gentilis* (Finland) | ♀ | 30 | 7.64 – 9.25 | 8.76 ± 0.06 |

**Table 8.** Modern and archaeological size ranges for *A. g. gentilis* ulna measurements. The *A. g. gentilis* data is made up of modern specimens from Norway, Sweden and Denmark. The *A. g. gentilis* (Finland) data represents only modern specimens from Finland (not including Lapland). The *A. gentilis* (Medieval) data is from Northern Goshawk recovered from Medieval dated contexts in Norway. Abbreviations: GL = greatest length, Dip = diagonal of the proximal end, Bp = breadth of the proximal end, Tp = depth of the proximal end, SC = smallest breadth of the corpus, Did = diagonal of the distal end. The measurements are in mm.

|  | **Sex** | **No. specimens** | **Observed range (mm)** | **Mean ± standard error** |
| --- | --- | --- | --- | --- |
| **Ulna GL** |  |  |  |  |
| *A. g. gentilis* (Norway & Sweden) | ♂ | 21 | 98.60 – 109.40 | 103.94 ± 0.51 |
| *A. g. gentilis* (Norway & Sweden) | ♀ | 7 | 113.50 – 121.15 | 116.43 ± 1.03 |
| *A. g. gentilis* (Denmark) | ♂ | 23 | 96.48 – 106.94 | 100.95 ± 0.58 |
| *A. g. gentilis* (Denmark) | ♀ | 21 | 110.66 – 121.41 | 115.24 ± 0.73 |
| *A. g. gentilis* (Finland) | ♂ | 22 | 98.97 – 108.73 | 104.90 ± 0.51 |
| *A. g. gentilis* (Finland) | ♀ | 30 | 112.61 – 121.94 | 117.49 ± 0.38 |
| *A. gentilis* (Medieval) | ♂ | 3 | 102.55 – 103.05 | 102.83 ± 0.15 |
| *A. gentilis* (Medieval) | ♀ | 4 | 117.89 – 122.04 | 120.23 ± 0.88 |
| **Ulna Dip** |  |  |  |  |
| *A. g. gentilis* (Norway & Sweden) | ♂ | 21 | 10.23 – 12.17 | 11.07 ± 0.11 |
| *A. g. gentilis* (Norway & Sweden) | ♀ | 8 | 11.90 – 13.18 | 12.50 ± 0.16 |
| *A. g. gentilis* (Denmark) | ♂ | 26 | 9.50 – 11.41 | 10.57 ± 0.11 |
| *A. g. gentilis* (Denmark) | ♀ | 21 | 11.34 – 13.40 | 12.32 ± 0.13 |
| *A. g. gentilis* (Finland) | ♂ | 22 | 10.61 – 12.15 | 11.33 ± 0.10 |
| *A. g. gentilis* (Finland) | ♀ | 30 | 12.30 – 13.66 | 12.93 ± 0.07 |
| *A. gentilis* (Medieval) | ♂ | 3 | 10.14 – 11.15 | 10.50 ± 0.32 |
| *A. gentilis* (Medieval) | ♀ | 5 | 11.89 – 13.55 | 12.73 ± 0.27 |
| **Ulna Bp** |  |  |  |  |
| *A. g. gentilis* (Norway & Sweden) | ♂ | 21 | 10.83 – 12.27 | 11.36 ± 0.07 |
| *A. g. gentilis* (Norway & Sweden) | ♀ | 8 | 12.41 – 12.91 | 12.72 ± 0.07 |
| *A. g. gentilis* (Denmark) | ♂ | 26 | 10.14 – 11.72 | 10.89 ± 0.08 |
| *A. g. gentilis* (Denmark) | ♀ | 21 | 12.01 – 13.77 | 12.80 ± 0.12 |
| *A. g. gentilis* (Finland) | ♂ | 22 | 10.87 – 11.92 | 11.44 ± 0.06 |
| *A. g. gentilis* (Finland) | ♀ | 30 | 12.25 – 14.26 | 13.31 ± 0.08 |
| *A. gentilis* (Medieval) | ♂ | 2 | 11.27 – 11.34 | 11.31 ± 0.04 |
| *A. gentilis* (Medieval) | ♀ | 5 | 12.59 – 13.61 | 13.16 ± 0.19 |
| **Ulna TP** |  |  |  |  |
| *A. g. gentilis* (Norway & Sweden) | ♂ | 21 | 8.17 – 9.12 | 8.61 ± 0.05 |
| *A. g. gentilis* (Norway & Sweden) | ♀ | 8 | 9.51 – 10.10 | 9.77 ± 0.08 |
| *A. g. gentilis* (Denmark) | ♂ | 26 | 7.75 – 8.99 | 8.28 ± 0.06 |
| *A. g. gentilis* (Denmark) | ♀ | 21 | 9.12 – 10.27 | 9.62 ± 0.08 |
| *A. g. gentilis* (Finland) | ♂ | 22 | 8.35 – 9.13 | 8.73 ± 0.04 |
| *A. g. gentilis* (Finland) | ♀ | 30 | 9.53 – 11.01 | 10.05 ± 0.05 |
| *A. gentilis* (Medieval) | ♂ | 2 | 8.34 – 8.39 | 8.37 ± 0.03 |
| *A. gentilis* (Medieval) | ♀ | 5 | 9.79 – 10.43 | 10.08 ± 0.11 |
| **Ulna SC** |  |  |  |  |
| *A. g. gentilis* (Norway & Sweden) | ♂ | 21 | 5.54 – 6.14 | 5.82 ± 0.04 |
| *A. g. gentilis* (Norway & Sweden) | ♀ | 8 | 6.02 – 7.07 | 6.43 ± 0.11 |
| *A. g. gentilis* (Denmark) | ♂ | 26 | 5.10 – 6.16 | 5.58 ± 0.05 |
| *A. g. gentilis* (Denmark) | ♀ | 21 | 5.95 – 7.18 | 6.47 ± 0.08 |
| *A. g. gentilis* (Finland) | ♂ | 22 | 5.48 – 6.25 | 5.82 ± 0.04 |
| *A. g. gentilis* (Finland) | ♀ | 30 | 6.11 – 7.12 | 6.56 ± 0.05 |
| *A. gentilis* (Medieval) | ♂ | 3 | 5.51 – 5.95 | 5.67 ± 0.14 |
| *A. gentilis* (Medieval) | ♀ | 5 | 6.36 – 6.74 | 6.56 ± 0.08 |
| **Ulna Did** |  |  |  |  |
| *A. g. gentilis* (Norway & Sweden) | ♂ | 21 | 9.76 – 11.17 | 10.22 ± 0.08 |
| *A. g. gentilis* (Norway & Sweden) | ♀ | 8 | 10.71 – 12.08 | 11.38 ± 0.15 |
| *A. g. gentilis* (Denmark) | ♂ | 26 | 9.11 – 10.47 | 9.74 ± 0.07 |
| *A. g. gentilis* (Denmark) | ♀ | 21 | 10.39 – 12.29 | 11.34 ± 0.13 |
| *A. g. gentilis* (Finland) | ♂ | 22 | 9.93 – 10.78 | 10.31 ± 0.05 |
| *A. g. gentilis* (Finland) | ♀ | 30 | 10.58 – 12.60 | 11.74 ± 0.07 |
| *A. gentilis* (Medieval) | ♂ | 3 | 10.01 – 10.29 | 10.12 ± 0.09 |
| *A. gentilis* (Medieval) | ♀ | 5 | 11.32 – 11.93 | 11.60 ± 0.11 |

**Table 9.** Modern and archaeological size ranges for *A. g. gentilis* carpometacarpus measurements. The *A. g. gentilis* data is made up of modern specimens from Norway, Sweden and Denmark. The *A. g. gentilis* (Finland) data represents only modern specimens from Finland (not including Lapland). The *A. gentilis* (Medieval) data is from Northern Goshawk recovered from Medieval dated contexts in Norway. Abbreviations: GL = greatest length, Bp = breadth of the proximal end, Did = diagonal of the distal end, HS = height of the symphysis (measurement taken from Otto, 1981). The measurements are in mm.

|  | **Sex** | **No. specimens** | **Observed range (mm)** | **Mean ± standard error** |
| --- | --- | --- | --- | --- |
| **Carpometacarpus GL** |  |  |  |  |
| *A. g. gentilis* (Norway & Sweden) | ♂ | 21 | 53.93 – 60.97 | 57.30 ± 0.33 |
| *A. g. gentilis* (Norway & Sweden) | ♀ | 8 | 62.56 – 67.11 | 63.92 ± 0.52 |
| *A. g. gentilis* (Denmark) | ♂ | 26 | 52.81 – 58.94 | 55.37 ± 0.31 |
| *A. g. gentilis* (Denmark) | ♀ | 19 | 60.60 – 67.21 | 63.16 ± 0.45 |
| *A. g. gentilis* (Finland) | ♂ | 22 | 55.62 – 60.59 | 57.83 ± 0.28 |
| *A. g. gentilis* (Finland) | ♀ | 30 | 62.22 – 67.10 | 65.22 ± 0.23 |
| *A. gentilis* (Medieval) | ♂ | 2 | 56.21 – 57.99 | 57.10 ± 0.89 |
| *A. gentilis* (Medieval) | ♀ | 3 | 64.71 – 67.48 | 65.97 ± 0.81 |
| **Carpometacarpus Bp** |  |  |  |  |
| *A. g. gentilis* (Norway & Sweden) | ♂ | 21 | 13.29 – 15.08 | 14.06 ± 0.09 |
| *A. g. gentilis* (Norway & Sweden) | ♀ | 8 | 15.00 – 16.36 | 15.80 ± 0.16 |
| *A. g. gentilis* (Denmark) | ♂ | 26 | 12.66 – 15.67 | 13.70 ± 0.12 |
| *A. g. gentilis* (Denmark) | ♀ | 19 | 15.17 – 16.96 | 15.84 ± 0.13 |
| *A. g. gentilis* (Finland) | ♂ | 21 | 13.72 – 14.97 | 14.27 ± 0.08 |
| *A. g. gentilis* (Finland) | ♀ | 30 | 15.69 – 17.93 | 16.50 ± 0.08 |
| *A. gentilis* (Medieval) | ♂ | 2 | 14.11 – 14.14 | 14.13 ± 0.02 |
| *A. gentilis* (Medieval) | ♀ | 4 | 15.83 – 16.67 | 16.36 ± 0.18 |
| **Carpometacarpus Did** | | |  |  |
| *A. g. gentilis* (Norway & Sweden) | ♂ | 21 | 8.01 – 9.70 | 8.59 ± 0.08 |
| *A. g. gentilis* (Norway & Sweden) | ♀ | 8 | 9.29 – 10.83 | 9.92 ± 0.20 |
| *A. g. gentilis* (Denmark) | ♂ | 26 | 7.76 – 9.89 | 8.58 ± 0.11 |
| *A. g. gentilis* (Denmark) | ♀ | 18 | 9.23 – 10.79 | 10.03 ± 0.10 |
| *A. g. gentilis* (Finland) | ♂ | 22 | 8.55 – 9.48 | 9.02 ± 0.06 |
| *A. g. gentilis* (Finland) | ♀ | 30 | 8.93 – 11.11 | 10.28 ± 0.08 |
| *A. gentilis* (Medieval) | ♂ | 2 | 9.32 – 9.38 | 9.35 ± 0.03 |
| *A. gentilis* (Medieval) | ♀ | 3 | 10.43 – 10.95 | 10.77 ± 0.17 |
| **Carpometacarpus HS** |  |  |  |  |
| *A. g. gentilis* (Norway & Sweden) | ♂ | 21 | 4.94 – 6.18 | 5.42 ± 0.07 |
| *A. g. gentilis* (Norway & Sweden) | ♀ | 8 | 5.68 – 6.97 | 6.21 ± 0.14 |
| *A. g. gentilis* (Denmark) | ♂ | 25 | 4.50 – 6.46 | 5.14 ± 0.09 |
| *A. g. gentilis* (Denmark) | ♀ | 19 | 5.41 – 7.47 | 6.26 ± 0.11 |
| *A. g. gentilis* (Finland) | ♂ | 22 | 4.91 – 5.73 | 5.27 ± 0.04 |
| *A. g. gentilis* (Finland) | ♀ | 30 | 5.71 – 7.19 | 6.34 ± 0.06 |
| *A. gentilis* (Medieval) | ♂ | 2 | 4.85 – 4.97 | 4.91 ± 0.06 |
| *A. gentilis* (Medieval) | ♀ | 3 | 6.34 – 7.08 | 6.73 ± 0.21 |

**Table 10.** Modern size ranges for *A. g. gentilis* pelvis measurements. The *A. g. gentilis* data is made up of modern specimens from Norway, Sweden and Denmark. The *A. g. gentilis* (Finland) data represents only modern specimens from Finland (not including Lapland). Abbreviations: GL = greatest length, LV = length along the vertebrae, CB = cranial breadth, SB = smallets breadth of the Partes glutaeae, AA = breadth between the borders of the acetabula, DiA = diameter of one acetabulum, BA = breadth across the two antitrochanter. The measurements are in mm.

|  | **Sex** | **No. specimens** | **Observed range (mm)** | **Mean ± standard error** |
| --- | --- | --- | --- | --- |
| **Pelvis GL** |  |  |  |  |
| *A. g. gentilis* (Norway & Sweden) | ♂ | 42 | 62.17 – 71.97 | 68.01 ± 0.34 |
| *A. g. gentilis* (Norway & Sweden) | ♀ | 25 | 75.24 – 82.34 | 79.40 ± 0.35 |
| *A. g. gentilis* (Denmark) | ♂ | 41 | 60.64 – 72.50 | 66.16 ± 0.47 |
| *A. g. gentilis* (Denmark) | ♀ | 26 | 71.72 – 80.62 | 75.88 ± 0.57 |
| *A. g. gentilis* (Finland) | ♂ | 12 | 62.98 – 70.70 | 67.96 ± 0.66 |
| *A. g. gentilis* (Finland) | ♀ | 20 | 73.14 – 83.40 | 79.15 ± 0.66 |
| **Pelvis LV** |  |  |  |  |
| *A. g. gentilis* (Norway & Sweden) | ♂ | 42 | 48.91 – 59.82 | 52.98 ± 0.38 |
| *A. g. gentilis* (Norway & Sweden) | ♀ | 25 | 54.27 – 64.06 | 60.74 ± 0.44 |
| *A. g. gentilis* (Denmark) | ♂ | 41 | 46.81 – 59.91 | 52.44 ± 0.35 |
| *A. g. gentilis* (Denmark) | ♀ | 26 | 54.44 – 63.28 | 60.00 ± 0.41 |
| *A. g. gentilis* (Finland) | ♂ | 12 | 46.23 – 56.51 | 52.75 ± 0.89 |
| *A. g. gentilis* (Finland) | ♀ | 21 | 56.86 – 64.06 | 60.70 ± 0.44 |
| **Pelvis CB** |  |  |  |  |
| *A. g. gentilis* (Norway & Sweden) | ♂ | 42 | 25.76 – 29.98 | 27.78 ± 0.17 |
| *A. g. gentilis* (Norway & Sweden) | ♀ | 25 | 29.41 – 35.85 | 32.26 ± 0.27 |
| *A. g. gentilis* (Denmark) | ♂ | 41 | 22.85 – 30.85 | 26.83 ± 0.28 |
| *A. g. gentilis* (Denmark) | ♀ | 27 | 26.37 – 33.99 | 30.20 ± 0.46 |
| *A. g. gentilis* (Finland) | ♂ | 12 | 27.42 – 30.25 | 28.49 ± 0.26 |
| *A. g. gentilis* (Finland) | ♀ | 18 | 29.15 – 35.17 | 32.61 ± 0.38 |
| **Pelvis SB** |  |  |  |  |
| *A. g. gentilis* (Norway & Sweden) | ♂ | 42 | 15.07 – 18.53 | 16.58 ± 0.13 |
| *A. g. gentilis* (Norway & Sweden) | ♀ | 25 | 16.91 – 20.54 | 18.94 ± 0.21 |
| *A. g. gentilis* (Denmark) | ♂ | 41 | 14.25 – 19.93 | 16.45 ± 0.16 |
| *A. g. gentilis* (Denmark) | ♀ | 27 | 15.72 – 21.24 | 18.48 ± 0.21 |
| *A. g. gentilis* (Finland) | ♂ | 12 | 14.66 – 18.47 | 17.00 ± 0.30 |
| *A. g. gentilis* (Finland) | ♀ | 20 | 17.33 – 19.98 | 18.86 ± 0.16 |
| **Pelvis AA** |  |  |  |  |
| *A. g. gentilis* (Norway & Sweden) | ♂ | 42 | 22.37 – 27.79 | 24.60 ± 0.16 |
| *A. g. gentilis* (Norway & Sweden) | ♀ | 25 | 25.91 – 30.18 | 28.24 ± 0.24 |
| *A. g. gentilis* (Denmark) | ♂ | 41 | 21.11 – 28.72 | 23.81 ± 0.23 |
| *A. g. gentilis* (Denmark) | ♀ | 26 | 24.07 – 29.10 | 26.75 ± 0.28 |
| *A. g. gentilis* (Finland) | ♂ | 12 | 22.73 – 27.39 | 24.99 ± 0.44 |
| *A. g. gentilis* (Finland) | ♀ | 20 | 26.24 – 31.29 | 28.03 ± 0.27 |
| **Pelvis DiA** |  |  |  |  |
| *A. g. gentilis* (Norway & Sweden) | ♂ | 42 | 6.45 – 7.80 | 6.94 ± 0.05 |
| *A. g. gentilis* (Norway & Sweden) | ♀ | 25 | 7.86 – 9.52 | 8.57 ± 0.08 |
| *A. g. gentilis* (Denmark) | ♂ | 41 | 6.02 – 7.53 | 6.82 ± 0.05 |
| *A. g. gentilis* (Denmark) | ♀ | 27 | 7.39 – 9.53 | 8.22 ± 0.11 |
| *A. g. gentilis* (Finland) | ♂ | 12 | 6.68 – 8.05 | 7.02 ± 0.11 |
| *A. g. gentilis* (Finland) | ♀ | 21 | 8.05 – 9.18 | 8.59 ± 0.08 |
| **Pelvis BA** |  |  |  |  |
| *A. g. gentilis* (Norway & Sweden) | ♂ | 42 | 34.50 – 39.80 | 36.90 ± 0.16 |
| *A. g. gentilis* (Norway & Sweden) | ♀ | 24 | 40.87 – 46.49 | 42.95 ± 0.29 |
| *A. g. gentilis* (Denmark) | ♂ | 41 | 32.79 – 42.31 | 36.04 ± 0.29 |
| *A. g. gentilis* (Denmark) | ♀ | 26 | 37.79 – 45.01 | 41.51 ± 0.38 |
| *A. g. gentilis* (Finland) | ♂ | 12 | 34.18 – 39.65 | 37.01 ± 0.49 |
| *A. g. gentilis* (Finland) | ♀ | 20 | 40.47 – 46.62 | 42.46 ± 0.35 |

**Table 11.** Modern and archaeological size ranges for *A. g. gentilis* femur measurements. The *A. g. gentilis* data is made up of modern specimens from Norway, Sweden and Denmark. The *A. g. gentilis* (Finland) data represents only modern specimens from Finland (not including Lapland). The *A. gentilis* (Medieval) data is from Northern Goshawk recovered from Medieval dated contexts in Norway. Abbreviations: GL = greatest length, Bp = breadth of the proximal end, Dp = depth of the proximal end, SC = smallest breadth of the corpus, Bd = breadth of the distal end, Dd = depth of the distal end. The measurements are in mm.

|  | **Sex** | **No. specimens** | **Observed range (mm)** | **Mean ± standard error** |
| --- | --- | --- | --- | --- |
| **Femur GL** |  |  |  |  |
| *A. g. gentilis* (Norway & Sweden) | ♂ | 57 | 73.30 – 82.13 | 78.40 ± 0.23 |
| *A. g. gentilis* (Norway & Sweden) | ♀ | 29 | 84.93 – 94.78 | 89.41 ± 0.40 |
| *A. g. gentilis* (Denmark) | ♂ | 49 | 71.88 – 82.63 | 76.83 ± 0.36 |
| *A. g. gentilis* (Denmark) | ♀ | 40 | 83.81 – 95.30 | 87.91 ± 0.42 |
| *A. g. gentilis* (Finland) | ♂ | 22 | 75.48 – 82.35 | 78.81 ± 0.38 |
| *A. g. gentilis* (Finland) | ♀ | 30 | 86.31 – 92.95 | 89.68 ± 0.30 |
| *A. gentilis* (Medieval) | ♂ | 2 | 78.27 – 78.50 | 78.39 ± 0.12 |
| *A. gentilis* (Medieval) | ♀ | 8 | 90.60 – 94.89 | 92.26 ± 0.49 |
| **Femur Bp** |  |  |  |  |
| *A. g. gentilis* (Norway & Sweden) | ♂ | 58 | 14.00 – 16.36 | 15.04 ± 0.07 |
| *A. g. gentilis* (Norway & Sweden) | ♀ | 30 | 17.14 – 19.28 | 18.02 ± 0.11 |
| *A. g. gentilis* (Denmark) | ♂ | 51 | 13.35 – 15.93 | 14.72 ± 0.09 |
| *A. g. gentilis* (Denmark) | ♀ | 41 | 15.68 – 19.18 | 17.77 ± 0.13 |
| *A. g. gentilis* (Finland) | ♂ | 21 | 14.43 – 16.66 | 15.30 ± 0.12 |
| *A. g. gentilis* (Finland) | ♀ | 30 | 16.81 – 19.51 | 18.41 ± 0.12 |
| *A. gentilis* (Medieval) | ♂ | 2 | 13.96 – 14.40 | 14.18 ± 0.22 |
| *A. gentilis* (Medieval) | ♀ | 10 | 16.80 – 19.19 | 18.26 ± 0.22 |
| **Femur Dp** |  |  |  |  |
| *A. g. gentilis* (Norway & Sweden) | ♂ | 58 | 7.72 – 9.40 | 8.66 ± 0.04 |
| *A. g. gentilis* (Norway & Sweden) | ♀ | 30 | 9.67 – 11.33 | 10.49 ± 0.08 |
| *A. g. gentilis* (Denmark) | ♂ | 51 | 7.54 – 9.29 | 8.34 ± 0.06 |
| *A. g. gentilis* (Denmark) | ♀ | 41 | 9.26 – 11.19 | 10.03 ± 0.08 |
| *A. g. gentilis* (Finland) | ♂ | 21 | 8.30 – 9.37 | 8.71 ± 0.06 |
| *A. g. gentilis* (Finland) | ♀ | 30 | 9.61 – 11.16 | 10.47 ± 0.07 |
| *A. gentilis* (Medieval) | ♂ | 2 | 7.95 – 8.48 | 8.22 ± 0.27 |
| *A. gentilis* (Medieval) | ♀ | 10 | 10.09 – 10.93 | 10.42 ± 0.08 |
| **Femur SC** |  |  |  |  |
| *A. g. gentilis* (Norway & Sweden) | ♂ | 57 | 6.27 – 7.67 | 6.98 ± 0.04 |
| *A. g. gentilis* (Norway & Sweden) | ♀ | 29 | 7.37 – 9.01 | 8.16 ± 0.06 |
| *A. g. gentilis* (Denmark) | ♂ | 49 | 5.87 – 7.79 | 6.83 ± 0.05 |
| *A. g. gentilis* (Denmark) | ♀ | 40 | 7.13 – 9.49 | 8.09 ± 0.08 |
| *A. g. gentilis* (Finland) | ♂ | 21 | 6.47 – 7.30 | 6.94 ± 0.05 |
| *A. g. gentilis* (Finland) | ♀ | 30 | 7.42 – 8.84 | 8.31 ± 0.06 |
| *A. gentilis* (Medieval) | ♂ | 2 | 7.19 – 7.35 | 7.27 ± 0.08 |
| *A. gentilis* (Medieval) | ♀ | 11 | 7.49 – 8.93 | 8.22 ± 0.12 |
| **Femur Bd** |  |  |  |  |
| *A. g. gentilis* (Norway & Sweden) | ♂ | 57 | 14.86 – 17.02 | 15.85 ± 0.06 |
| *A. g. gentilis* (Norway & Sweden) | ♀ | 29 | 17.89 – 20.40 | 19.38 ± 0.12 |
| *A. g. gentilis* (Denmark) | ♂ | 50 | 14.38 – 17.35 | 15.34 ± 0.09 |
| *A. g. gentilis* (Denmark) | ♀ | 40 | 16.91 – 20.63 | 18.73 ± 0.13 |
| *A. g. gentilis* (Finland) | ♂ | 21 | 14.93 – 16.64 | 16.01 ± 0.09 |
| *A. g. gentilis* (Finland) | ♀ | 30 | 17.60 – 20.37 | 19.54 ± 0.11 |
| *A. gentilis* (Medieval) | ♂ | 2 | 15.65 – 15.94 | 15.80 ± 0.15 |
| *A. gentilis* (Medieval) | ♀ | 9 | 18.80 – 20.19 | 19.53 ± 0.16 |
| **Femur Dd** |  |  |  |  |
| *A. g. gentilis* (Norway & Sweden) | ♂ | 57 | 9.84 – 11.67 | 10.70 ± 0.06 |
| *A. g. gentilis* (Norway & Sweden) | ♀ | 29 | 12.22 – 13.93 | 13.10 ± 0.09 |
| *A. g. gentilis* (Denmark) | ♂ | 50 | 9.46 – 11.50 | 10.32 ± 0.06 |
| *A. g. gentilis* (Denmark) | ♀ | 40 | 11.64 – 14.22 | 12.66 ± 0.09 |
| *A. g. gentilis* (Finland) | ♂ | 21 | 10.27 – 11.59 | 10.80 ± 0.08 |
| *A. g. gentilis* (Finland) | ♀ | 30 | 12.33 – 13.96 | 13.25 ± 0.08 |
| *A. gentilis* (Medieval) | ♂ | 2 | 10.66 – 11.55 | 11.11 ± 0.45 |
| *A. gentilis* (Medieval) | ♀ | 9 | 12.65 – 14.23 | 13.49 ± 0.16 |

**Table 12.** Modern and archaeological size ranges for *A. g. gentilis* tibiotarsus measurements. The *A. g. gentilis* data is made up of modern specimens from Norway, Sweden and Denmark. The *A. g. gentilis* (Finland) data represents only modern specimens from Finland (not including Lapland). The *A. gentilis* (Medieval) data is from Northern Goshawk recovered from Medieval dated contexts in Norway. Abbreviations: GL = greatest length, Dip = diagonal of the proximal end (defined as, from the Condylus medialis femoralis to the Crista lateralis), Bp = breadth of the proximal end, SC = smallest breadth of the corpus, Bd = breadth of the distal end, Dd = depth of the distal end. The measurements are in mm.

|  | **Sex** | **No. specimens** | **Observed range (mm)** | **Mean ± standard error** |
| --- | --- | --- | --- | --- |
| **Tibiotarsus GL** |  |  |  |  |
| *A. g. gentilis* (Norway & Sweden) | ♂ | 23 | 97.48 – 107.22 | 103.42 ± 0.46 |
| *A. g. gentilis* (Norway & Sweden) | ♀ | 9 | 113.13 – 121.22 | 116.14 ± 0.86 |
| *A. g. gentilis* (Denmark) | ♂ | 25 | 96.70 – 106.07 | 101.31 ± 0.48 |
| *A. g. gentilis* (Denmark) | ♀ | 23 | 111.63 – 122.31 | 116.29 ± 0.62 |
| *A. g. gentilis* (Finland) | ♂ | 22 | 98.34 – 107.41 | 103.89 ± 0.49 |
| *A. g. gentilis* (Finland) | ♀ | 30 | 113.15 – 120.94 | 117.69 ± 0.36 |
| *A. gentilis* (Medieval) | ♂ | 0 | - | - |
| *A. gentilis* (Medieval) | ♀ | 10 | 115.29 – 123.28 | 119.72 ± 0.81 |
| **Tibiotarsus Dip** |  |  |  |  |
| *A. g. gentilis* (Norway & Sweden) | ♂ | 23 | 14.36 – 16.10 | 15.29 ± 0.10 |
| *A. g. gentilis* (Norway & Sweden) | ♀ | 10 | 17.71 – 19.26 | 18.35 ± 0.14 |
| *A. g. gentilis* (Denmark) | ♂ | 27 | 13.99 – 16.37 | 14.78 ± 0.10 |
| *A. g. gentilis* (Denmark) | ♀ | 23 | 17.25 – 19.82 | 18.34 ± 0.17 |
| *A. g. gentilis* (Finland) | ♂ | 22 | 14.29 – 16.37 | 15.45 ± 0.10 |
| *A. g. gentilis* (Finland) | ♀ | 30 | 17.56 – 19.73 | 19.05 ± 0.09 |
| *A. gentilis* (Medieval) | ♂ | 0 | - | - |
| *A. gentilis* (Medieval) | ♀ | 9 | 18.20 – 19.64 | 18.92 ± 0.18 |
| **Tibiotarsus Bp** |  |  |  |  |
| *A. g. gentilis* (Norway & Sweden) | ♂ | 18 | 10.84 – 11.87 | 11.39 ± 0.07 |
| *A. g. gentilis* (Norway & Sweden) | ♀ | 10 | 12.96 – 14.95 | 13.61 ± 0.21 |
| *A. g. gentilis* (Denmark) | ♂ | 27 | 10.31 – 11.88 | 11.00 ± 0.08 |
| *A. g. gentilis* (Denmark) | ♀ | 23 | 12.91 – 15.37 | 13.62 ± 0.15 |
| *A. g. gentilis* (Finland) | ♂ | 22 | 9.73 – 12.30 | 11.04 ± 0.13 |
| *A. g. gentilis* (Finland) | ♀ | 30 | 12.79 – 14.99 | 13.68 ± 0.10 |
| *A. gentilis* (Medieval) | ♂ | 0 | - | - |
| *A. gentilis* (Medieval) | ♀ | 7 | 13.48 – 15.09 | 14.36 ± 0.18 |
| **Tibiotarsus SC** |  |  |  |  |
| *A. g. gentilis* (Norway & Sweden) | ♂ | 23 | 5.64 – 6.49 | 6.23 ± 0.05 |
| *A. g. gentilis* (Norway & Sweden) | ♀ | 9 | 6.78 – 7.68 | 7.09 ± 0.10 |
| *A. g. gentilis* (Denmark) | ♂ | 26 | 5.48 – 6.67 | 5.97 ± 0.06 |
| *A. g. gentilis* (Denmark) | ♀ | 23 | 6.52 – 8.19 | 7.18 ± 0.11 |
| *A. g. gentilis* (Finland) | ♂ | 22 | 5.73 – 6.90 | 6.31 ± 0.07 |
| *A. g. gentilis* (Finland) | ♀ | 30 | 6.76 – 7.90 | 7.41 ± 0.05 |
| *A. gentilis* (Medieval) | ♂ | 1 | 5.85 | 5.85 ± 0 |
| *A. gentilis* (Medieval) | ♀ | 15 | 6.33 – 8.64 | 7.51 ± 0.13 |
| **Tibiotarsus Bd** |  |  |  |  |
| *A. g. gentilis* (Norway & Sweden) | ♂ | 23 | 11.22 – 12.41 | 11.80 ± 0.07 |
| *A. g. gentilis* (Norway & Sweden) | ♀ | 9 | 13.40 – 14.47 | 13.98 ± 0.11 |
| *A. g. gentilis* (Denmark) | ♂ | 26 | 10.93 – 12.39 | 11.53 ± 0.08 |
| *A. g. gentilis* (Denmark) | ♀ | 23 | 13.45 – 15.39 | 14.23 ± 0.11 |
| *A. g. gentilis* (Finland) | ♂ | 22 | 11.11 – 12.62 | 11.92 ± 0.09 |
| *A. g. gentilis* (Finland) | ♀ | 30 | 13.85 – 15.08 | 14.56 ± 0.05 |
| *A. gentilis* (Medieval) | ♂ | 1 | 11.52 | 11.52 ± 0 |
| *A. gentilis* (Medieval) | ♀ | 16 | 12.92 – 15.36 | 14.65 ± 0.14 |
| **Tibiotarsus Dd** |  |  |  |  |
| *A. g. gentilis* (Norway & Sweden) | ♂ | 23 | 7.83 – 8.64 | 8.25 ± 0.05 |
| *A. g. gentilis* (Norway & Sweden) | ♀ | 9 | 9.36 – 10.00 | 9.66 ± 0.08 |
| *A. g. gentilis* (Denmark) | ♂ | 26 | 7.52 – 8.85 | 8.01 ± 0.07 |
| *A. g. gentilis* (Denmark) | ♀ | 23 | 9.14 – 10.74 | 9.77 ± 0.09 |
| *A. g. gentilis* (Finland) | ♂ | 22 | 7.67 – 8.85 | 8.28 ± 0.06 |
| *A. g. gentilis* (Finland) | ♀ | 30 | 9.44 – 10.96 | 10.11 ± 0.05 |
| *A. gentilis* (Medieval) | ♂ | 1 | 7.86 | 7.86 ± 0 |
| *A. gentilis* (Medieval) | ♀ | 13 | 9.47 – 10.65 | 10.08 ± 0.08 |

**Table 13.** Modern and archaeological size ranges for *A. g. gentilis* tarsometatarsus measurements. The *A. g. gentilis* data is made up of modern specimens from Norway, Sweden and Denmark. The *A. g. gentilis* (Finland) data represents only modern specimens from Finland (not including Lapland). The *A. gentilis* (Medieval) data is from Northern Goshawk recovered from Medieval dated contexts in Norway. Abbreviations: GL = greatest length, Bp = breadth of the proximal end, SC = smallest breadth of the corpus, Bd = breadth of the distal end. The measurements are in mm.

|  | **Sex** | **No. specimens** | **Observed range (mm)** | **Mean ± standard error** |
| --- | --- | --- | --- | --- |
| **Tarsometatarsus GL** |  |  |  |  |
| *A. g. gentilis* (Norway & Sweden) | ♂ | 21 | 71.91 – 79.06 | 76.48 ± 0.46 |
| *A. g. gentilis* (Norway & Sweden) | ♀ | 10 | 82.50 – 88.34 | 84.42 ± 0.60 |
| *A. g. gentilis* (Denmark) | ♂ | 25 | 70.37 – 79.32 | 74.95 ± 0.43 |
| *A. g. gentilis* (Denmark) | ♀ | 21 | 81.22 – 89.02 | 84.57 ± 0.47 |
| *A. g. gentilis* (Finland) | ♂ | 22 | 73.13 – 80.37 | 77.27 ± 0.39 |
| *A. g. gentilis* (Finland) | ♀ | 30 | 81.63 – 89.92 | 86.29 ± 0.31 |
| *A. gentilis* (Medieval) | ♂ | 3 | 73.08 – 79.01 | 75.20 ± 1.91 |
| *A. gentilis* (Medieval) | ♀ | 8 | 84.43 – 89.50 | 87.12 ± 0.57 |
| **Tarsometatarsus Bp** |  |  |  |  |
| *A. g. gentilis* (Norway & Sweden) | ♂ | 22 | 11.97 – 13.24 | 12.71 ± 0.07 |
| *A. g. gentilis* (Norway & Sweden) | ♀ | 10 | 14.32 – 15.51 | 14.96 ± 0.13 |
| *A. g. gentilis* (Denmark) | ♂ | 25 | 11.51 – 13.39 | 12.22 ± 0.11 |
| *A. g. gentilis* (Denmark) | ♀ | 21 | 13.32 – 16.40 | 14.92 ± 0.17 |
| *A. g. gentilis* (Finland) | ♂ | 22 | 12.04 – 13.74 | 12.76 ± 0.09 |
| *A. g. gentilis* (Finland) | ♀ | 30 | 14.62 – 16.31 | 15.43 ± 0.08 |
| *A. gentilis* (Medieval) | ♂ | 2 | 11.98 – 12.40 | 12.19 ± 0.21 |
| *A. gentilis* (Medieval) | ♀ | 12 | 13.54 – 16.41 | 15.31 ± 0.22 |
| **Tarsometatarsus SC** |  |  |  |  |
| *A. g. gentilis* (Norway & Sweden) | ♂ | 21 | 4.73 – 5.75 | 5.18 ± 0.06 |
| *A. g. gentilis* (Norway & Sweden) | ♀ | 10 | 5.87 – 7.04 | 6.28 ± 0.13 |
| *A. g. gentilis* (Denmark) | ♂ | 26 | 4.80 – 5.93 | 5.15 ± 0.06 |
| *A. g. gentilis* (Denmark) | ♀ | 20 | 5.97 – 7.67 | 6.65 ± 0.10 |
| *A. g. gentilis* (Finland) | ♂ | 22 | 4.48 – 5.93 | 5.33 ± 0.07 |
| *A. g. gentilis* (Finland) | ♀ | 30 | 6.12 – 7.40 | 6.69 ± 0.06 |
| *A. gentilis* (Medieval) | ♂ | 3 | 4.97 – 6.00 | 5.39 ± 0.31 |
| *A. gentilis* (Medieval) | ♀ | 15 | 5.01 – 7.40 | 6.48 ± 0.15 |
| **Tarsometatarsus Bd** |  |  |  |  |
| *A. g. gentilis* (Norway & Sweden) | ♂ | 22 | 11.80 – 13.86 | 12.84 ± 0.09 |
| *A. g. gentilis* (Norway & Sweden) | ♀ | 10 | 15.02 – 16.32 | 15.45 ± 0.15 |
| *A. g. gentilis* (Denmark) | ♂ | 25 | 11.89 – 13.12 | 12.43 ± 0.07 |
| *A. g. gentilis* (Denmark) | ♀ | 20 | 13.96 – 17.38 | 15.38 ± 0.17 |
| *A. g. gentilis* (Finland) | ♂ | 22 | 12.19 – 13.71 | 12.96 ± 0.10 |
| *A. g. gentilis* (Finland) | ♀ | 30 | 14.98 – 16.58 | 15.89 ± 0.07 |
| *A. gentilis* (Medieval) | ♂ | 2 | 11.80 – 12.89 | 12.35 ± 0.55 |
| *A. gentilis* (Medieval) | ♀ | 13 | 12.85 – 17.07 | 15.79 ± 0.30 |
