# Supplementary material for: A long‐term study of size variation in Northern Goshawk Accipiter gentilis across Scandinavia, with a focus on Norway
Source: Ecol Evol. 2023 Dec 7;13(12):e10789. doi: 10.1002/ece3.10789 (PMC10701624; doi:10.1002/ece3.10789)
Supplement: Supplementary file 4 — File S4. [file ECE3-13-e10789-s003.docx]

**Supporting Information File 4 (SIF4)**. PCA results.

**Table 1.** PCAs for all males and females. Modern and archaeological males compared in analysis 1, 3, 5, 7, 9, 11. Modern and archaeological females compared in analysis 2, 4, 6, 8, 10, 12. Important to note that not all measurements were available for some of the archaeological specimens. Specimens with more than 2 measurements missing were not included within the PCA to prevent skewing of the results.

| **Component** | **Eigenvalue** | **% Variance** | **Loadings** | **PC1** | **PC2** | **PC3** | **PC4** | **PC5** | **PC6** |
| --- | --- | --- | --- | --- | --- | --- | --- | --- | --- |
| *Analysis 1, Humerus PCA results ♂, n = 93 (Modern n = 91, Archaeological n = 2)* | | | | | | | | | |
| **PC1** | 7.331 | 94.91 | GL | 0.950 | -0.308 | -0.039 | 0.036 | -0.003 | - |
| **PC2** | 0.291 | 3.76 | Bp | 0.228 | 0.790 | -0.545 | 0.159 | 0.041 | - |
| **PC3** | 0.052 | 0.67 | SC | 0.088 | 0.276 | 0.633 | 0.705 | -0.132 | - |
| **PC4** | 0.030 | 0.39 | Bd | 0.182 | 0.427 | 0.475 | -0.676 | -0.317 | - |
| **PC5** | 0.021 | 0.27 | KB | 0.067 | 0.148 | 0.274 | -0.136 | 0.938 | - |
| *Analysis 2, Humerus PCA results ♀, n = 54 (Modern n = 45, Archaeological n = 9)* | | | | | | | | | |
| **PC1** | 9.843 | 93.93 | GL | 0.945 | -0.320 | -0.055 | -0.015 | -0.027 | - |
| **PC2** | 0.457 | 4.36 | Bp | 0.230 | 0.753 | -0.535 | 0.299 | 0.069 | - |
| **PC3** | 0.099 | 0.94 | SC | 0.103 | 0.192 | 0.639 | 0.657 | -0.337 | - |
| **PC4** | 0.062 | 0.59 | Bd | 0.192 | 0.535 | 0.436 | -0.683 | -0.141 | - |
| **PC5** | 0.019 | 0.18 | KB | 0.077 | 0.085 | 0.336 | 0.112 | 0.928 | - |
| *Analysis 3, Ulna PCA results ♂, n = 50 (Modern n = 47, Archaeological n = 3)* | | | | | | | | | |
| **PC1** | 8.195 | 95.38 | GL | 0.971 | -0.212 | -0.102 | -0.021 | -0.009 | -0.030 |
| **PC2** | 0.238 | 2.77 | Dip | 0.132 | 0.845 | -0.490 | 0.083 | 0.079 | -0.120 |
| **PC3** | 0.083 | 0.96 | Bp | 0.121 | 0.292 | 0.594 | 0.417 | -0.531 | -0.303 |
| **PC4** | 0.038 | 0.45 | Tp | 0.096 | 0.208 | 0.172 | 0.170 | -0.074 | 0.940 |
| **PC5** | 0.025 | 0.29 | SC | 0.054 | 0.032 | 0.305 | 0.490 | 0.809 | -0.093 |
| **PC6** | 0.013 | 0.16 | Did | 0.113 | 0.333 | 0.523 | -0.742 | 0.228 | -0.029 |
| *Analysis 4, Ulna PCA results ♀, n = 33 (Modern n = 29, Archaeological n = 4)* | | | | | | | | | |
| **PC1** | 11.831 | 95.77 | GL | 0.976 | -0.207 | -0.045 | 0.040 | 0.007 | -0.019 |
| **PC2** | 0.351 | 2.84 | Dip | 0.105 | 0.672 | -0.718 | 0.066 | 0.073 | -0.107 |
| **PC3** | 0.096 | 0.78 | Bp | 0.108 | 0.397 | 0.465 | -0.282 | 0.697 | -0.222 |
| **PC4** | 0.044 | 0.36 | Tp | 0.089 | 0.245 | 0.067 | -0.398 | -0.081 | 0.873 |
| **PC5** | 0.020 | 0.16 | SC | 0.050 | 0.303 | 0.332 | 0.848 | 0.029 | 0.274 |
| **PC6** | 0.011 | 0.09 | Did | 0.118 | 0.443 | 0.389 | -0.190 | -0.708 | -0.319 |
| *Analysis 5, Carpometacarpus PCA results ♂, n = 49 (Modern n = 47, Archaeological n = 2)* | | | | | | | | |  |
| **PC1** | 3.549 | 89.16 | GL | 0.960 | -0.204 | -0.022 | -0.191 | - | - |
| **PC2** | 0.221 | 5.54 | Bp | 0.234 | 0.357 | -0.353 | 0.833 | - | - |
| **PC3** | 0.129 | 3.25 | Did | 0.105 | 0.897 | -0.031 | -0.428 | - | - |
| **PC4** | 0.082 | 2.06 | HS | 0.114 | 0.160 | 0.935 | 0.295 | - | - |
| *Analysis 6, Carpometacarpus PCA results ♀, n = 30 (Modern n = 27, Archaeological n = 3)* | | | | | | | | |  |
| **PC1** | 4.062 | 90.87 | GL | 0.956 | -0.141 | -0.245 | 0.081 | - | - |
| **PC2** | 0.186 | 4.16 | Bp | 0.217 | 0.320 | 0.387 | -0.837 | - | - |
| **PC3** | 0.169 | 3.78 | Did | 0.158 | -0.269 | 0.884 | 0.347 | - | - |
| **PC4** | 0.053 | 1.19 | HS | 0.120 | 0.897 | 0.088 | 0.415 | - | - |
| *Analysis 7, Femur PCA results ♂, n = 108 (Modern n = 106, Archaeological n = 2)* | | | | | | | | | |
| **PC1** | 5.580 | 89.56 | GL | 0.944 | -0.317 | -0.076 | -0.009 | -0.049 | 0.026 |
| **PC2** | 0.300 | 4.82 | Bp | 0.176 | 0.707 | -0.425 | -0.306 | -0.437 | 0.066 |
| **PC3** | 0.180 | 2.89 | Dp | 0.127 | 0.325 | 0.051 | 0.833 | -0.117 | -0.410 |
| **PC4** | 0.065 | 1.04 | SC | 0.074 | 0.234 | 0.008 | 0.358 | 0.279 | 0.857 |
| **PC5** | 0.056 | 0.89 | Bd | 0.209 | 0.445 | 0.178 | -0.250 | 0.763 | -0.286 |
| **PC6** | 0.049 | 0.79 | Dd | 0.116 | 0.202 | 0.883 | -0.149 | -0.364 | 0.107 |
| *Analysis 8, Femur PCA results ♀, n = 77 (Modern n = 69, Archaeological n = 8)* | | | | | | | | | |
| **PC1** | 8.439 | 89.53 | GL | 0.926 | -0.369 | -0.060 | -0.034 | -0.041 | 0.001 |
| **PC2** | 0.528 | 5.60 | Bp | 0.196 | 0.612 | -0.186 | -0.714 | -0.205 | -0.015 |
| **PC3** | 0.178 | 1.89 | Dp | 0.134 | 0.345 | 0.008 | 0.439 | -0.432 | 0.695 |
| **PC4** | 0.138 | 1.46 | SC | 0.098 | 0.215 | -0.403 | 0.075 | 0.814 | 0.338 |
| **PC5** | 0.073 | 0.77 | Bd | 0.232 | 0.531 | -0.018 | 0.527 | 0.032 | -0.620 |
| **PC6** | 0.070 | 0.74 | Dd | 0.151 | 0.206 | 0.894 | -0.111 | 0.326 | 0.131 |
| *Analysis 9, Tibiotarsus PCA results ♂ – insufficient archaeological data* | | | | | | | | | |
| **PC1** | - | - | GL | - | - | - | - | - | - |
| **PC2** | - | - | Dip | - | - | - | - | - | - |
| **PC3** | - | - | Bp | - | - | - | - | - | - |
| **PC4** | - | - | SC | - | - | - | - | - | - |
| **PC5** | - | - | Bd | - | - | - | - | - | - |
| **PC6** | - | - | Dd | - | - | - | - | - | - |
| *Analysis 10, Tibiotarsus PCA results ♀, n = 41 (Modern n = 32, Archaeological n = 9)* | | | | | | | | | |
| **PC1** | 10.747 | 93.54 | GL | 0.958 | -0.282 | -0.044 | -0.010 | -0.008 | -0.010 |
| **PC2** | 0.472 | 4.11 | Dip | 0.165 | 0.616 | -0.376 | -0.527 | -0.066 | -0.412 |
| **PC3** | 0.148 | 1.29 | Bp | 0.161 | 0.399 | 0.883 | -0.165 | 0.057 | 0.058 |
| **PC4** | 0.065 | 0.57 | SC | 0.096 | 0.354 | -0.086 | 0.624 | 0.636 | -0.254 |
| **PC5** | 0.029 | 0.26 | Bd | 0.109 | 0.417 | -0.075 | 0.528 | -0.707 | 0.174 |
| **PC6** | 0.028 | 0.24 | Dd | 0.086 | 0.287 | -0.251 | -0.165 | 0.297 | 0.855 |
| *Analysis 11, Tarsometatarsus PCA results ♂, n = 49 (Modern n = 46, Archaeological n = 3)* | | | | | | | | | |
| **PC1** | 5.273 | 93.47 | GL | 0.985 | -0.162 | 0.055 | -0.040 | - | - |
| **PC2** | 0.238 | 4.21 | Bp | 0.114 | 0.848 | 0.089 | -0.509 | - | - |
| **PC3** | 0.087 | 1.55 | SC | 0.030 | 0.286 | 0.737 | 0.612 | - | - |
| **PC4** | 0.043 | 0.77 | Bd | 0.130 | 0.415 | -0.668 | 0.604 | - | - |
| *Analysis 12, Tarsometatarsus PCA results ♀, n = 44 (Modern n = 30, Archaeological n = 14)* | | | | | | | | | |
| **PC1** | 4.582 | 85.45 | GL | 0.951 | -0.306 | -0.031 | -0.001 | - | - |
| **PC2** | 0.451 | 8.42 | Bp | 0.213 | 0.588 | 0.741 | -0.244 | - | - |
| **PC3** | 0.179 | 3.34 | SC | 0.084 | 0.248 | 0.095 | 0.960 | - | - |
| **PC4** | 0.150 | 2.79 | Bd | 0.206 | 0.706 | -0.664 | -0.134 | - | - |
|  |  |  |  |  |  |  |  |  |  |
