# Supplementary material for: A long‐term study of size variation in Northern Goshawk Accipiter gentilis across Scandinavia, with a focus on Norway
Source: Ecol Evol. 2023 Dec 7;13(12):e10789. doi: 10.1002/ece3.10789 (PMC10701624; doi:10.1002/ece3.10789)
Supplement: Supplementary file 5 — File S5. [file ECE3-13-e10789-s004.docx]

**Supporting Information File 5 (SIF5)**. Size range data for the two Viking Age *Accipiter gentilis* specimens recovered from the Gokstad ship burial. Diagrams and detailed descriptions of how these measurements were taken can be found in Von den Driesch (1976), unless noted in the caption.

**Table 1. S**ize ranges for the Viking Age Gokstad *A. gentilis* bones. Abbreviations: GL = greatest length, Bp = breadth of the proximal end, SC = smallest breadth of the corpus, Bd = breadth of the distal end, KB = smallest depth of the distal shaft (measurement taken from Kraft, 1972), Dip = diagonal of the proximal end, Tp = depth of the proximal end, Did = diagonal of the distal end. The measurements are in mm.

| **Element/measurement** | **Sex** | **No. specimens** | **Observed range (mm)** | **Mean ± standard error** |
| --- | --- | --- | --- | --- |
| **Humerus GL** |  |  |  |  |
| *A. gentilis* (Viking) | ♀ | 2 | 104.16 – 105.17 | 104.67 ± 0.51 |
| **Humerus Bp** |  |  |  |  |
| *A. gentilis* (Viking) | ♀ | 2 | 23.38 – 23.50 | 23.44 ± 0.06 |
| **Humerus SC** |  |  |  |  |
| *A. gentilis* (Viking) | ♀ | 3 | 7.89 – 8.07 | 7.97 ± 0.05 |
| **Humerus Bd** |  |  |  |  |
| *A. gentilis* (Viking) | ♀ | 2 | 19.59 – 19.78 | 19.69 ± 0.10 |
| **Humerus KB** |  |  |  |  |
| *A. gentilis* (Viking) | ♀ | 2 | 7.13 – 7.19 | 7.16 ± 0.03 |
| **Ulna GL** |  |  |  |  |
| *A. gentilis* (Viking) | ♀ | 0 | - | - |
| **Ulna Dip** |  |  |  |  |
| *A. gentilis* (Viking) | ♀ | 0 | - | - |
| **Ulna Bp** |  |  |  |  |
| *A. gentilis* (Viking) | ♀ | 0 | - | - |
| **Ulna TP** |  |  |  |  |
| *A. gentilis* (Viking) | ♀ | 0 | - | - |
| **Ulna SC** |  |  |  |  |
| *A. gentilis* (Viking) | ♀ | 2 | 5.95 – 6.25 | 6.10 ± 0.15 |
| **Ulna Did** |  |  |  |  |
| *A. gentilis* (Viking) | ♀ | 1 | 11.40 | 11.40 ± 0 |
| **Femur GL** |  |  |  |  |
| *A. gentilis* (Viking) | ♀ | 3 | 88.65 – 89.15 | 88.90 ± 0.14 |
| **Femur Bp** |  |  |  |  |
| *A. gentilis* (Viking) | ♀ | 3 | 17.33 – 17.69 | 17.52 ± 0.10 |
| **Femur Dp** |  |  |  |  |
| *A. gentilis* (Viking) | ♀ | 3 | 9.52 – 10.03 | 9.73 ± 0.15 |
| **Femur SC** |  |  |  |  |
| *A. gentilis* (Viking) | ♀ | 4 | 7.55 – 8.06 | 7.82 ± 0.12 |
| **Femur Bd** |  |  |  |  |
| *A. gentilis* (Viking) | ♀ | 4 | 15.56 – 18.98 | 17.59 ± 0.72 |
| **Femur Dd** |  |  |  |  |
| *A. gentilis* (Viking) | ♀ | 4 | 11.82 – 12.91 | 12.50 ± 0.25 |
| **Tibiotarsus GL** |  |  |  |  |
| *A. gentilis* (Viking) | ♀ | 1 | 117.23 | 117.23 ± 0 |
| **Tibiotarsus Dip** |  |  |  |  |
| *A. gentilis* (Viking) | ♀ | 1 | 17.50 | 17.50 ± 0 |
| **Tibiotarsus Bp** |  |  |  |  |
| *A. gentilis* (Viking) | ♀ | 0 | - | - |
| **Tibiotarsus SC** |  |  |  |  |
| *A. gentilis* (Viking) | ♀ | 1 | 6.64 | 6.64 ± 0 |
| **Tibiotarsus Bd** |  |  |  |  |
| *A. gentilis* (Viking) | ♀ | 1 | 13.65 | 13.65 ± 0 |
| **Tibiotarsus Dd** |  |  |  |  |
| *A. gentilis* (Viking) | ♀ | 1 | 8.87 | 8.87 ± 0 |
| **Tarsometatarsus GL** |  |  |  |  |
| *A. gentilis* (Viking) | ♀ | 1 | 83.56 | 83.56 ± 0 |
| **Tarsometatarsus Bp** |  |  |  |  |
| *A. gentilis* (Viking) | ♀ | 2 | 14.16 – 14.58 | 14.37 ± 0.21 |
| **Tarsometatarsus SC** |  |  |  |  |
| *A. gentilis* (Viking) | ♀ | 1 | 5.87 | 5.87 ± 0 |
| **Tarsometatarsus Bd** |  |  |  |  |
| *A. gentilis* (Viking) | ♀ | 0 | - | - |

**Figure 1.** Viking Age Gokstad *Accipiter gentilis* females plotted in relation to the Norwegian Medieval specimens and modern specimens from Norway, Sweden and Denmark.

**
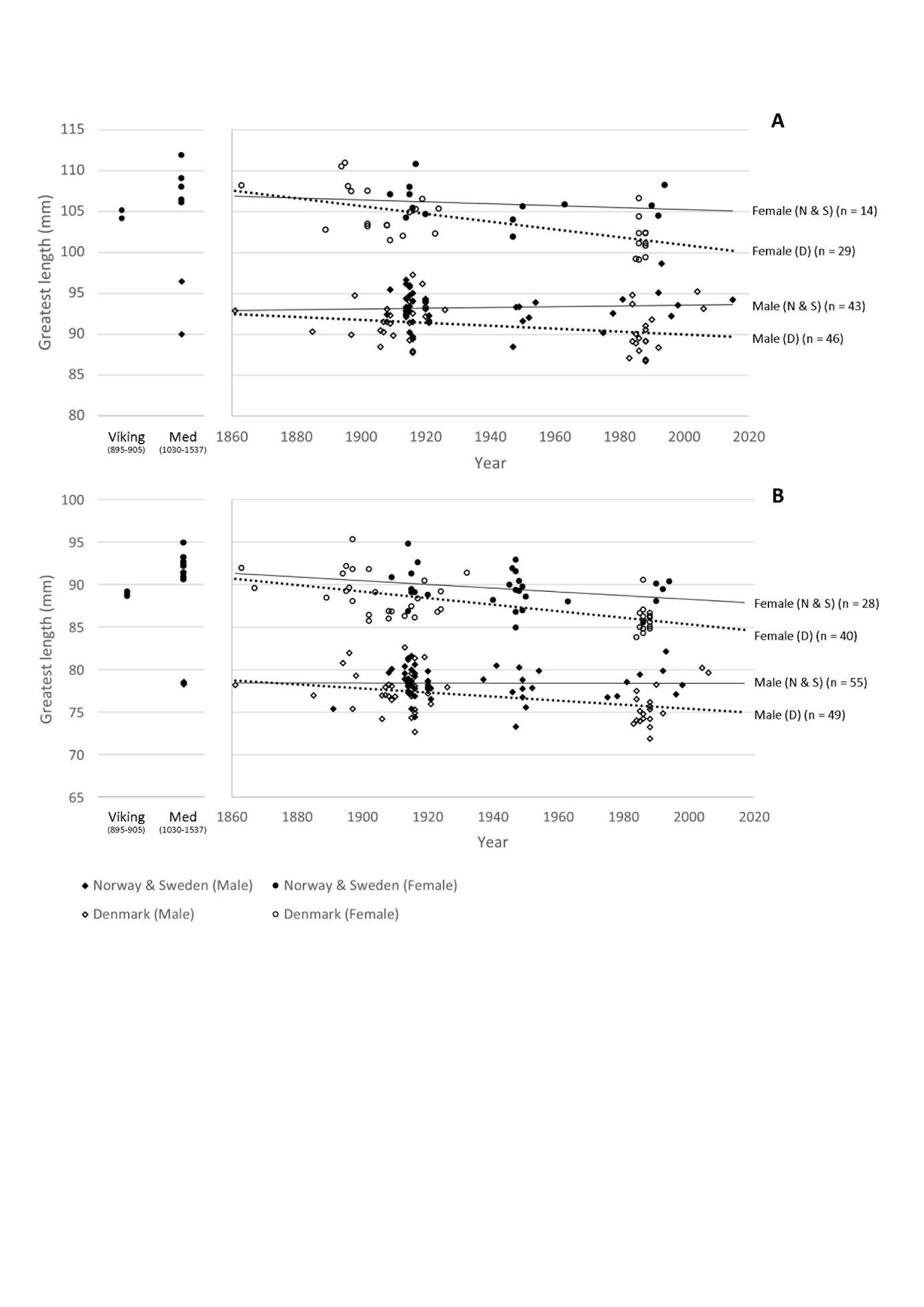
**
